# Gestational immune activation disrupts hypothalamic neurocircuits of maternal care behavior

**DOI:** 10.1038/s41380-022-01602-x

**Published:** 2022-05-17

**Authors:** Alice Zambon, Laura Cuenca Rico, Mathieu Herman, Anna Gundacker, Amina Telalovic, Lisa-Marie Hartenberger, Rebekka Kuehn, Roman A. Romanov, S. Abid Hussaini, Tibor Harkany, Daniela D. Pollak

**Affiliations:** 1 Department of Neurophysiology and Neuropharmacology, Center for Physiology and Pharmacology, Medical University of Vienna, Vienna, Austria; 2 Department of Pathology and Cell Biology, Taub Institute, Columbia University Irving Medical Center, New York, NY USA; 3 Department of Molecular Neurosciences, Center for Brain Research, Medical University of Vienna, Vienna, Austria

**Keywords:** Neuroscience, Psychiatric disorders

## Abstract

Immune activation is one of the most common complications during pregnancy, predominantly evoked by viral infections. Nevertheless, how immune activation affects mother–offspring relationships postpartum remains unknown. Here, by using the polyinosinic-polycytidylic acid (Poly I:C) model of gestational infection we show that viral-like immune activation at mid-gestation persistently changes hypothalamic neurocircuit parameters in mouse dams and, consequently, is adverse to parenting behavior. Poly I:C-exposed dams favor non-pup-directed exploratory behavior at the expense of pup retrieval. These behavioral deficits are underlain by dendrite pruning and lesser immediate early gene activation in Galanin (Gal)^+^ neurons with dam-specific transcriptional signatures that reside in the medial preoptic area (mPOA). Reduced activation of an exclusively inhibitory contingent of these distal-projecting Gal^+^ neurons allows for increased feed-forward inhibition onto putative dopaminergic neurons in the ventral tegmental area (VTA) in Poly I:C-exposed dams. Notably, destabilized VTA output specifically accompanies post-pup retrieval epochs. We suggest that gestational immunogenic insults bias both threat processing and reward perception, manifesting as disfavored infant caregiving.

## Introduction

During gestation, the female physiology undergoes substantial adaptations to ensure optimal pregnancy outcomes and to prepare the future mother for the demands of caretaking of their young after birth. In altricial species, including humans and most rodents, the newborn is critically dependent on parental care. Adequate parental care is not only imperative to ensure survival, feeding, and protection. Instead, brain development of the altricial young continues after birth and is shaped by the early environment, on which parental care has the strongest programming impact during the initial postnatal phase.

In the vast majority of mammals, including laboratory mice, parenting is organized in a uniparental system with the female exclusively providing offspring care [1, 2]. Maternal care comprises a repertoire of goal-directed behaviors ultimately aimed at both nurturing and safeguarding the offspring. While individual behavioral displays are species-specific, the neural substrates of maternal care are evolutionarily conserved across mammals [3]. Although hardwired neural circuits subserve maternal care, which is instinctively and spontaneously displayed in the presence of the offspring [1, 2, 4], a high degree of plasticity enables the integration of endogenous and exogenous stimuli for the regulation of behavioral displays [5–7]. Nevertheless, major gaps in our knowledge exist on how adverse environmental stimuli affect stereotypic behaviors, particularly maternal care behavior.

The first evidence for an effect of maternal immune activation (MIA) on postpartum maternal factors is provided by cross-fostering studies in which being reared by an immune-challenged surrogate confers risk for distinct forms of psychopathology in the adult life of affected offspring [8, 9]. Therefore, and considering that prenatal infection is a major vulnerability factor for the development of psychiatric disorders in adolescent or adult offspring [10–14], the lack of information on neuronal consequences of MIA in the mother’s brain and how potential changes in circuit wiring weigh in on maternal-to-offspring bonding and nursing postpartum, is surprising.

To address these questions, we have selected the polyinosinic:polycytidylic acid (Poly I:C) model of MIA because it faithfully recapitulates the pathobiology of maternal viral-like gestational infection, and since circumstantial evidence alludes to probable modifications of maternal care behavior in this model [15–18]. Here, we provide a comprehensive characterization of MIA-induced behavioral deficits in mothers postpartum, which we associate with persistent morphological changes in neurons of the medial preoptic area (mPOA) of the rostral hypothalamus, the central regulatory hub for the neural organization of maternal care [4, 19, 20]. We show that MIA specifically reduces immediate early gene expression in galanin (Gal)^+^ neurons in the mPOA, which project to the ventral tegmental area (VTA). We then seek to establish causality between reduced synaptic signaling in this mPOA-to-VTA pathway and altered maternal care behavior because of a stereotypical antagonistic change in the activity of putative GABAergic and putative dopaminergic VTA neurons, coincident with behavioral epochs of pup retrieval in Poly I:C-exposed mothers. Overall, our data highlight the importance of closely surveying the consequences of viral infection during pregnancy on human maternal behavior, maternal mental health, and the incidence of parenting disorders, given that pregnant women may be more susceptible to and/or more strongly affected by infectious diseases, especially respiratory viruses [21, 22].

## Materials and methods

A detailed description of all procedures can be found in the supplementary materials and methods chapter and in Supplementary Tables 1–4. Additional information about sample sizes and complete statistical probing of our data are reported in Supplementary Tables 5–8.

### Animals

Animal experiments were conducted following the ARRIVE ethical guidelines and the U.K. Animals Scientific Procedures Act, 1986 and associated guidelines (EU Directive 2010/63/EU for animal experiments). The national ethical committee of animal care and use approved all animal procedures and experiments (2020–0.193.053 and AC-AAAS1500 for in vivo electrophysiology). Animals were housed under standard conditions. Adult (2–5 months) C57Bl/6 N female mice were used for all wildtype (WT) experiments, including MIA behavioral studies, Golgi–COX staining, and single-unit in vivo electrophysiology. Transgenic lines were (BAC)Gal::Cre, FlexEYFP, vGat::Flp, and vGlut::Flp mice (Supplementary Table 2). Gal::Cre males were bred with homozygous FlexEYFP females to obtain Gal::Cre//FlexEYFP female mice. Gal::Cre and Gal::Cre//FlexEYFP female mice were then used respectively for viral procedures and c-Fos staining upon MIA. vGat::Flp//Gal::Cre and vGlut::Flp//Gal::Cre females were obtained by crossing Gal::Cre females and respectively vGat::Flp and vGlut::Flp males and used for viral tracing.

### Maternal immune activation (MIA)

A timed mating procedure was applied for all MIA experiments as previously described [17]. Poly I:C was obtained from Sigma (P9582) and injected at embryonic day (ED) 12.5 at a concentration of 20 mg/kg. Pregnant females received either 10 μL/g of Poly I:C or vehicle solution (0.9% NaCl) i.p. All experiments were conducted following the standard conditions suggested by Kentner and colleagues [23] and as previously described by our laboratory [15–17, 24]. Detailed MIA information is reported in Supplementary Table 3. Sickness behavior was tested 2 h after Poly I:C vs. vehicle injection in pregnant mice and all mice were checked for weight loss 24 h after immune activation. Mice that did not show both weight loss and apparent sickness behavior were excluded from the analysis.

### Behavioral experiments

#### Pup retrieval assay

The pup retrieval assay was conducted on postnatal day (PD) 4 under dim light conditions (19–20 Lux) following a standard procedure [20, 25] and analyzed by the EthoVision XT software (Noldus, Wageningen, the Netherlands).

#### Ultrasonic vocalizations (USVs)

USVs were recorded and analyzed at PD4. Four pups were randomly selected from each litter and tested using an USB Ultrasound microphone (Petterson, Ultravox system). Parameters considered were the duration of calls, amplitude (represented as linear amplitude), the frequency at maximum peak and the total number of calls per litter. Calls were further divided into short, flat, chevron, complex, upward and downward categories and analyzed based as published earlier [26] (Supplementary Table 1).

### Golgi–Cox and Nissl stainings

The FD Rapid GolgiStain^TM^ Kit (FD Neurotechnologies) was used for the Golgi–Cox staining procedure for neuronal reconstruction of neurons in the mPOA (bregma + 0.26 and −0.22) and in the VTA (bregma −2.92 and −3.88) of PD7 and nulliparous control Poly I:C- and vehicle-treated females following the manufacturer’s instructions.

#### Neurolucida reconstruction and analysis

Neurolucida 10 (MBF Bioscience) was used for the reconstruction of neuronal morphologies in the mPOA and in the VTA of Golgi–Cox-stained brains. In the mPOA, neuronal morphology was analyzed from 4 to 5 multipolar neurons (Bregma + 0.14 and 0.02) per mouse (*N* = 6 mice/group).

The morphology of six neurons was reconstructed in the VTA (bregma −2.92 and −3.28) of each Golgi–Cox stained brain (*N* = 5 mice/group). The number of spines was counted in four segments of VTA primary dendrites and secondary dendrites per brain hemisphere. Spines were classified as stubby, mushroom, thin, long thin and filopodia based on the algorithm provided by ref. [27].

### Viral tracing

#### Viruses

Information on all viruses used is summarized in Supplementary Table 2. The anterograde adenoviruses (AAVs) AAV-FLEx-syn1-EGFP and AAV-FRT-Ef1a-tdTomato were stereotactically injected into the mPOA of vGat::Flp//Gal::Cre and vGlut::Flp//Gal::Cre females. The retrograde adenoviruses (AAVrg) AAVrg-FLEx-CAG-tdTomato, AAVrg-FLEx-hsyn-EGFP and AAVrg-FRT-mCherry were stereotactically injected into the VTA of Gal::Cre, vGat::Flp//Gal::Cre and vGlut::Flp//Gal::Cre females.

#### Stereotactic surgery for virus injection

Deep anesthesia was maintained using isoflurane (2%, 1 L/min flow rate; Forane) and the head of each mouse was fixed in a stereotactic frame (Model 1900, David Kopf Instruments, Tujunga, CA, USA). AAV-FLEx-syn1-EGFP and AAV-FRT-Ef1a-tdTomato were mixed and injected in the left hemisphere of the mPOA of vGat::Flp//Gal::Cre and vGlut::Flp//Gal::Cre females using a 30° angle (coordinates: Anterior/Posterior (AP) = + 0.3, Medio/Lateral (ML) = −2.6, Dorso/Ventral (DV) = −5.0). AAVrg-FLEx-CAG-tdTomato was injected in the left hemisphere of the VTA of *Gal::Cre* females (coordinates: AP = −3.0, ML = −0.5, DV = −4.6), while AAVrg-FLEx-hsyn-EGFP and AAVrg-FRT-Ef1a-mCherry were mixed and injected in the left hemisphere of the VTA of vGat::Flp//Gal::Cre and vGlut::Flp//Gal::Cre females (coordinates: AP = −3.0, ML = −0.5, DV = −4.6). Viral expression was evaluated 4 weeks after injection.

### Phospho-c-Fos (c-Fos) analysis

For histochemical analysis of immediate early gene expression, Gal::Cre//FlexEYFP females were sacrificed 90 min after pup retrieval test.

#### Perfusion and fluorescence immunohistochemistry

Mice were transcardially perfused with 20 mL phosphate-buffered saline solution (1x PBS, pH 7.4) followed by 20 mL paraformaldehyde (PFA, 4%) in PBS.

Brains were cryosectioned at 30-µm thickness coronally. For c-Fos detection in Gal::Cre//FlexEYFP female mice, sections containing the mPOA (between bregma +0.02 and −0.1) were selected. Primary antibodies were anti-phospho-c-Fos (D82C12, Cell Signalling Technology; 1:8000) and anti-GFP (ab13970, Abcam, 1:800) raised in rabbit (monoclonal) and chicken, respectively. Secondary antibodies included Alexa Fluor™ 647-conjugated goat anti-rabbit 1:600 (A-21244, Invitrogen) and Alexa Fluor 488-conjugated goat anti-chicken 1:500 (A-11039, Invitrogen). Specimens were routinely incubated in DAPI 1:1000 (D9542, Merck) 5 min before mounting.

For tracing and morphological experiments in Gal::Cre, vGat::Flp//Gal::Cre, and vGlut::Flp//Gal::Cre females, sections spanning the mPOA and VTA (bregma + 0.02 and −0.1, bregma −2.92 and −3.16, respectively) were immunostained. Primary antibodies used were anti-GFP (ab290, Abcam, 1:800), anti-mCherry (CPCA-mCherry, EnCor Biotechnology, 1:1 000) and anti-TH (MAB318, Merck Millipore, 1:500). Secondary antibodies included Alexa Fluor 488-conjugated goat anti-rabbit (A-11008, Invitrogen, 1:500), Alexa Fluor 555-conjugated goat anti-chicken (ab150170, Abcam, 1:500) and Alexa Fluor 647-conjugated donkey anti-mouse (A-32787, Invitrogen, 1:500).

#### Imaging, cell counting, and analysis

Sections were imaged using a Nikon A1 laser-scanning microscope using the NIS-Elements AR software (version 5.02.01, Nikon Instruments, Tokyo, JP) at ×63 or ×20 primary magnification. Confocal images were collected at a resolution of 512 × 512 pixels with orthogonal steps of 2 µm (mPOA) or 0.5 µm (VTA) in all laser lines (405, 488, 564, and 647 nm). The QuPath software [28] was used for cell counting in the mPOA and the vBNST. ImageJ-1.53c was used for anatomical characterization.

### Single-unit in vivo electrophysiology recording

#### Surgery and tetrode implantation

In all, 16-channel microdrives were constructed as previously described [29, 30]. In all, 3–5 month-old pregnant females were used for single-unit in vivo electrophysiology. Females were implanted at ED15, after being either injected with a vehicle or Poly I:C solution. Tetrode implantation was conducted as described [31, 32]. Coordinates for VTA were AP = −3.2, ML = −0.6, DV = −4–4.2.

#### In vivo recording and single-unit analysis

Females were habituated to handling and attaching pre-amplifiers to the implanted microdrives for 10 min every day from PD0-3. At PD4, baseline recording was performed for 10 min followed by 15 min of the pup retrieval assay with four foster pups. Single units were recorded using the Axona DacqUSB system (Axona, UK) [31, 32]. Offline spike sorting was done using the Tint cluster-cutting software and KlustaKwik automated clustering, followed by further manual cleaning. Sorted units were processed to determine neuronal spike widths, firing rates, bursting using custom-built software (SpikeAnalysisGUI, SpikeWidthGUI, Spiketimesplot and RasterGUI https://github.com/HussainiLab available upon request; and FRATE from Axona).

#### Classification of neurons

A total of 136 VTA neurons were recorded from ten mice (Vehicle, *Ν* = 60; Poly I:C, *Ν* = 76). Neurons were first divided into fast-firing and slow-firing types based on their baseline average firing rate (FR). Neurons with FR > 10 Hz were classified as fast-faring, while neurons with FR < 10 Hz were classified as slow-firing.

Based on the frequency distribution histogram of pooled single-unit spike widths, narrow-spiking neurons were considered as neurons having spike width <1.2 ms and wide-spiking neurons as ones having spike width >1.4 ms. Bursting percentage (%) was measured from wide-spiking neurons. Bursts were defined as having two spikes with an interspike interval (ISI) < 80 ms and total burst duration not exceeding 160 ms [33].

### Bioinformatics of open-label single-cell RNA-sequencing data

**Fig. 1:**
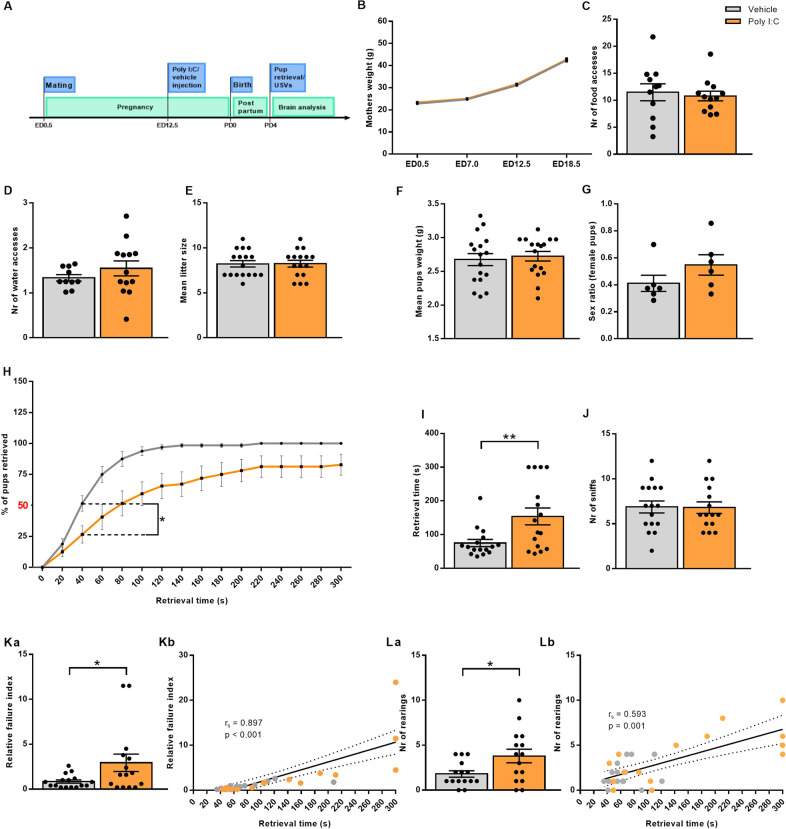
Deficient maternal behavior after gestational Poly I:C injection (MIA). **A** Schematic depiction of the experimental timeline. **B**–**G** MIA is not affecting **B** mothers’ weight gain during pregnancy (*N* = 16 animals/group), **C** food (*N* = 10–11 animals/group) and **D** water (*N* = 12–13 animals/group) access during lactation. No effect of MIA on **E** litter size (*N* = 16–17 litters/group), **F** average weight of pups at PD4 (*N* = 17 litters/group), **G** sex ratio per litter *(N* = 6 litters/group). **H** Cumulative retrieval in control versus Poly I:C-treated mothers indicating the time point of statistical analysis was conducted (*U* = 64.5; *Ρ* = 0.015; *Ν* = 16 animals/group). **I** Latency to retrieve all pups (*t*_(20.290)_ = −2.891; *Ρ* = 0.009; *Ν* = 16 animals/group). **J** MIA is not affecting the number of sniffs. Number of sniffs was measured in the first 30 s after the dam was introduced in the cage. **K** MIA affects retrieval accuracy. **Ka** Relative failure index is higher in MIA dams compared to controls (*t*_(29)_=−2.192; *Ρ* = 0.037; *Ν* = 15–16 animals/group). **Kb** Retrieval time significantly correlates with relative failure index (*r*_s_ = 0.897; *Ρ* < 0.001; *Ν* = 32 animals). **L** Increased exploratory behavior in MIA mothers, represented by number of rearings in the first minute after the mother was introduced in the cage: **La** Number of rearings is higher in Poly I:C-treated than control dams (*t*_*(*19.858)_ = −2.392; *Ρ* = 0.027; *Ν* = 15 animals/group) and **Lb** number of rearings correlates with retrieval time (*r*_s_ = 0.593; *Ρ* = 0.001; *Ν* = 30 animals). All data are presented as mean ± SEM, *Ρ  <  0.05, **Ρ < 0.01.

Single-cell RNA-sequencing data were reprocessed from GSE113576 [34]. To focus on Gal^+^ clusters, we extracted a subset of cells containing the original clusters i8, i16, i18 localized to the mPOA (Figs. 1C and 2A in ref. [34]). The Seurat3 package was used to compute gene expression in these clusters, as per the original source paper. Genes selected for expression analysis were: *Il6r*, *Il1r1*, *Jak1/2*, *Stat1-6*, *Ifnar1/2*, *Ifngr1/2*, *Tlr3/4*, *Myd88*, *Ticam1*, *Tirap*, *Mal*, *Tram*, *Traf3/6*, *Ikbkb*, *Chuk*, *nfk-b1/2*, and *mapk14*. Data are presented as dot plots (Fig. 4G). Males and females were analyzed separately.

**Fig. 2:**
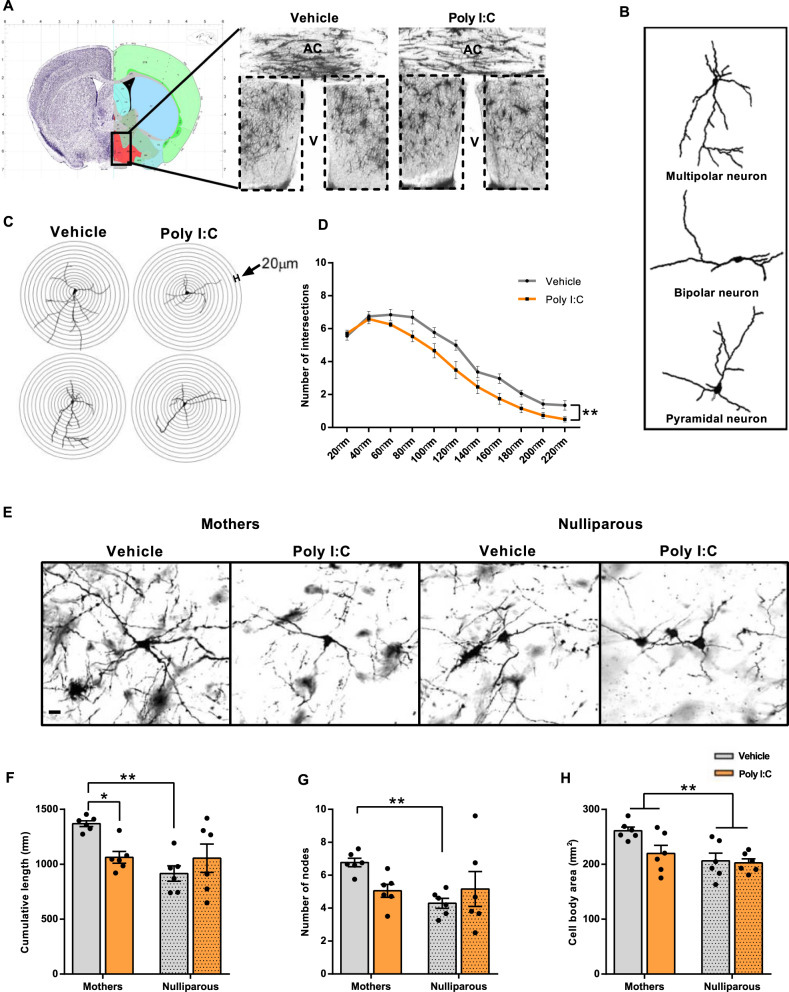
Altered morphology of mPOA multipolar neurons in postpartum females after gestational Poly I:C injection. **A** Schematic representation of the mPOA in coronal section (adapted from Allen Brain Atlas, Bregma 0.02) and magnification of Golgi–Cox impregnated mPOA region from control and Poly I:C-treated mothers (×1 magnification; AC anterior commissure, V ventricle). Dashed squares indicate the mPOA region. **B** Neurolucida reconstruction of identified populations of neurons: multipolar, pyramidal, and bipolar. **C**, **D** Sholl analysis: **C** Example of neuronal reconstructions for Sholl analysis in multipolar neurons from Poly I:C and vehicle-injected mothers (*r* = 20 µm; *r*1 = r + 20 µm; *r*2 = r1 + 20 µm etc.). **D** Poly I:C-injected mothers show significant lower number of intersections with concentric circles (main effect of treatment *F*_(1)_ = 10.511; *Ρ* = 0.009; *Ν* = 6 animals/group). **E** Examples of Golgi–Cox impregnated mPOA multipolar neurons to illustrate morphological differences between vehicle and Poly I:C-injected mothers and nulliparous females (20X magnification; scale bar: 20 µm). **F** Cumulative dendritic length is significantly reduced in neurons of the mPOA in Poly I:C-injected postpartum females (mothers), but not nulliparous females in comparison to vehicle injection (significant interaction of pregnancy and treatment: *F*_(1)_ = 7.888; *Ρ* = 0.011; *Ν* = 6 animals/group). **G** Significant increase in the number of dendritic nodes of mPOA multipolar neurons in mothers as compared to nulliparous controls is abolished in Poly I:C-injected females (significant interaction of pregnancy and treatment: *F*_(1)_ = 4.654; *Ρ* = 0.043; *Ν* = 6 animals/group). **H** Cell body area is significantly bigger in mPOA multipolar neurons in mothers irrespective of treatment (main effect of pregnancy: *F*_(1)_ = 10.180; *Ρ* = 0.005; *Ν* = 6 animals/group). Neuronal morphology was analyzed in 4–5 multipolar neurons from two sections per animal, with a total of 24–30 neurons per group. All data are presented as mean ± SEM, *P < 0.05, **Ρ < 0.01.

### Statistics

All analyzes were performed by an investigator blinded to the experimental treatment of mice. Sample sizes were selected based upon previous experience in the laboratory and common practice based on data available in the literature [20, 25, 35]. Statistical outliers were calculated with the online available Graphpad outlier calculator, using α = 0.05 as the level of significance. Mann–Whitney *U* test, Student’s *t* test and Spearman correlation were used for all behavioral analysis and for phospho-c-Fos histochemistry. Statistical assessment of neuronal morphology and spine density was based upon two-way analysis of variance (ANOVA). Mixed ANOVA was applied for Sholl analysis on log-transformed data. In vivo electrophysiology data were analyzed with Student’s *t* test, mixed ANOVA or the Mann–Whitney *U* test when data were not normally distributed. Graphs were made in GraphPad Prism 7 (San Diego, CA, USA).

## Results

### MIA disrupts maternal caretaking behavior postpartum

To determine the impact of viral-like gestational infection on maternal behavior, MIA was induced at ED12.5 in pregnant mice by the viral-mimic Poly I:C (20 mg/kg, i.p.) [15–18, 24] (Fig. 1A). Poly I:C-treated and control mothers were equivalent in weight gain during pregnancy (Fig. 1B) and in accessing food and water between PD1 and PD6, excluding any effect of MIA on the general metabolic function of the dam (Fig. 1C, D). Moreover, MIA did not impact the number of pups per litter (Fig. 1E), their weight on PD4 (Fig. 1F), and the sex ratio within litters (Fig. 1G).

Spontaneous maternal behaviors such as pup licking and grooming seem affected by Poly I:C treatment [15, 16]. However, the effects of MIA on either maternal responsivity or motivation [36] are unknown. To address this question, we used the pup retrieval assay and found a significantly reduced percentage of pups retrieved by mothers injected with Poly I:C during pregnancy (Fig. 1H), as compared to control mothers that had on average already retrieved 50% of the pups 40 s after the beginning of the test (Supplementary Videos 1 and 2). Correspondingly, MIA dams displayed an increased latency or failure to retrieve all pups back into their nest within 5 min (Fig. 1I), evidencing their reduced motivation to protect their offspring and inefficient care behavior. In order to exclude that this increased latency was due to a deficiency in locating/recognizing the pups due to potential Poly I:C-induced sensory impairment to the mother, we calculated the number of times the mother sniffed her pups. No significant difference between the groups was detected (Fig. 1J), suggesting that MIA mothers were aware of the presence and location of their offspring. We next asked whether the increased latency was only due to reduced motivation to retrieve and inefficient retrieval and/or decreased accuracy in their retrieval behavior. To address the latter, we analyzed the number of times the mother had approached the pups and moved back to the nest without retrieving or dropping them along the way (termed as “relative failure index”). Poly I:C-injected mothers approached without retrieving/dropped the pups more frequently than controls did (Fig. 1Ka). A significant positive correlation was also found between the retrieval time and the relative failure index, indicating that the increased latency was likely due to a reduced accuracy of maternal conduct (Fig. 1Kb).

Next, we asked if occupation with non-pup-related behaviors could prevent MIA dams from retrieving their pups more rapidly and accurately. We evaluated rearing behavior, a form of non-pup-directed exploration aimed at surveying the environment [37, 38]. MIA dams showed an increased number of rearing events as compared to controls, indicating their engagement in non-pup-related exploration at the expense of prioritizing the safety of the isolated pups (Fig. 1La). The number of rearing events significantly correlated with the retrieval time, indicating that higher engagement in rearing activity may offset pup-related behavior (Fig. 1Lb). Collectively, these observations demonstrate that MIA mothers are inefficient and inaccurate in providing pup-directed care and increasingly engage in stochastic environmental exploration instead.

### MIA does not affect offspring ultrasonic vocalizations

Mother-pup relationships are critically modulated by the pups’ ability to call out to their mothers. For this, pups typically use ultrasonic vocalizations (USVs), which are triggered by isolation from the mother and the nest and are aimed at the dams to efficiently locate their offspring. Accordingly, USVs imminently evoke pup retrieval responses [39] (Supplementary Fig. 1A). To evaluate the possibility that MIA could bias maternal behavior by impairing the pups’ ability of USVs, we recorded pup USVs prior to their retrieval to test if the retrieval latency of MIA dams would be increased. Isolated pups from Poly I:C-injected dams did not display any USV deficits, as compared to controls in call amplitude (Supplementary Fig. 1B), duration (Supplementary Fig. 1C), frequency (Supplementary Fig. 1D), and number of calls per litter (Supplementary Fig. 1E). Given that USVs are organized in sequences of different call types [40], we also determined if MIA specifically impacted on any of the 6 categories known [26] (Supplementary Table 1). No significant difference was found in either the mean amplitude, duration, frequency, or number of calls per litter amongst the Chevron, Complex, Flat, Downward, Upward and Short call sequences. The only statistical exception was an increased duration of “Flat” calls from MIA pups, which constitute simple constant frequency calls (Supplementary Fig. 1F–K), and whose increased frequency is against our null hypothesis of reduced USVs bringing about dysfunctional pup-to-mother communication. Thus, we suggest that the communicative behavior of MIA pups did not bias maternal caretaking.

### Neuronal morphology in the maternal mPOA is altered by gestational immune activation

Next, we sought to define if long-lasting changes in neuronal structure and function underpin maternal behavioral deficits postpartum upon MIA during pregnancy. We hypothesized that the mPOA could be particularly susceptible because its lesion completely disrupts pup retrieval [19, 41] and since both pregnancy and the postpartum period alter neural plasticity within this hypothalamic area [35, 42]. First, we gained insights in detailed neuronal morphology by using single-cell reconstructions after Golgi–Cox histochemistry of mPOA-containing specimens [42]. Our choice of the Golgi–Cox method was facilitated by data on the altered dendritic complexity of mPOA neurons in dams actively nursing and lactating their young [35]. By convention [43], we have distinguished pyramidal, bipolar, and multipolar neuronal subsets in the mPOA and compared their morphologies in MIA vs. control mothers (Fig. 2A, B). We found no difference between MIA and control mothers in either the pyramidal or the bipolar subpopulation of mPOA neurons for any of the parameters tested (Supplementary Fig. 2A–F). In contrast, for multipolar neurons in MIA mothers, Sholl analysis along both proximal and distal dendritic branches at 20-µm resolution (that is, radius (*r*) = 20 µm; *r*_1_ = *r* + 20 µm; *r*_2_ = *r*_1_ + 20 µm etc. Fig. 2C) exhibited significantly reduced numbers of dendritic intersections along the entire length of their dendritic trees (Fig. 2D). Next, we asked if MIA also affected cumulative dendritic length, the number of nodes and the cell body area, critical predictors of synaptic information transfer [44, 45]. In comparison to nulliparous females, vehicle-treated mothers had an increased cumulative length of the dendritic tree, confirming previous findings on structural plasticity being associated with maternal physiology in the mPOA upon pregnancy and/or care for the offspring [35]. Notwithstanding, Poly I:C treatment completely abolished these structural modifications (Fig. 2E, F). We made the same observation for the number of nodes (Fig. 2E, G). The size of the cell soma was significantly bigger in offspring-nursing vs. nulliparous females, but was not affected by MIA (Fig. 2H), which we interpreted as a lack of a general increase in cellular activity upon MIA in postpartum females. These results suggest that gestational Poly I:C treatment renders the female brain vulnerable by disrupting pregnancy-related neuronal remodeling, which is required for the development of maternal responsiveness to pups [46].

### Reduced c-Fos expression in Gal^+^ neurons during pup retrieval by MIA mothers

Spatial-transcriptomics [34] and single-cell transcriptomics place the Gal-containing neuronal cohort as the largest within the mPOA, including >40% of Gal^+^ neurons during brain development [47] and 20% stably expressing Gal in the adult brain [20]. Gal is an inducible neuropeptide with up to >1000-fold mRNA transcript increases upon stimuli [48], amongst which parenting behaviors are noted as well [49]. Genetic ablation of maternal Gal^+^ neurons reduces pup retrieval [20]. In contrast, optogenetic stimulation of Gal^+^ neurons in males suppresses pup-directed aggression [20]. Given the ample evidence available in implicating Gal^+^ neurons in parental physiology and goal-oriented behavior, we posited that this very neuronal contingent, particularly if they fell into the class of multipolar neurons, could be linked to dysfunction maternal behavior postpartum.

**Fig. 3:**
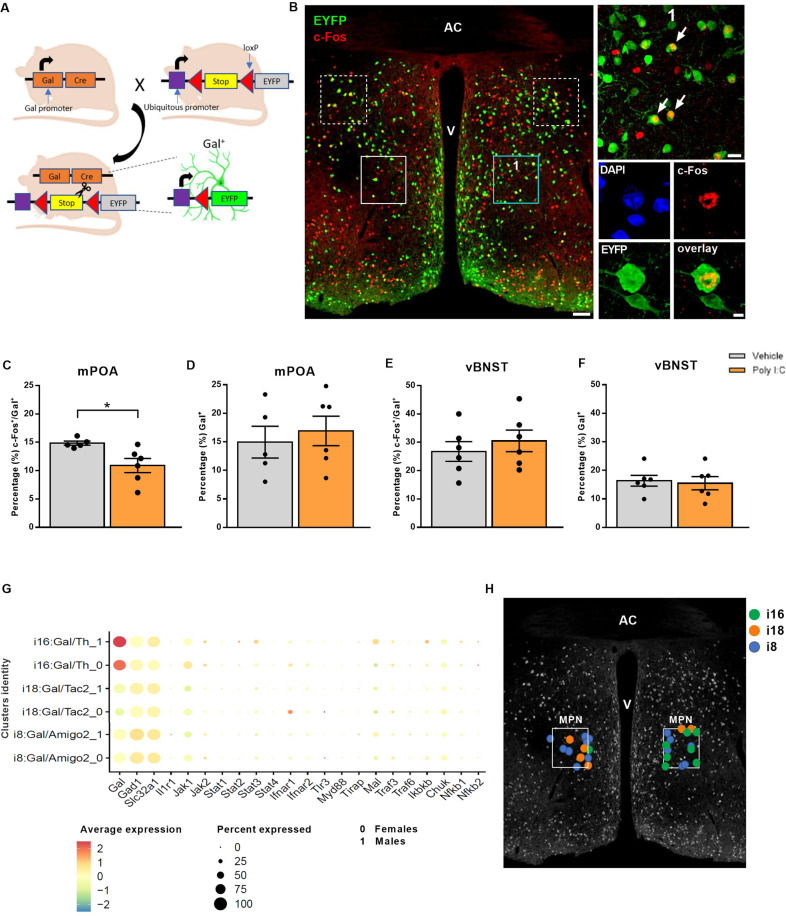
Reduced activation of mPOA Gal^+^ cells during pup retrieval in MIA females. **A** Breeding strategy for the generation of Gal::Cre//FlexEYFP mice. **B** Representative section of the mPOA (4 × 3 stitched 20X image; scale bar: 100 µm; AC anterior commissure, V ventricle) used for the evaluation of neuronal activity during pup retrieval in MIA and control dams. Boxes mark the bilateral ROIs within the mPOA (continuous line) or within the vBNST (dashed line) where c-Fos immunoreactivity was evaluated; the turquois box indicates the magnified area shown in (1) (60X magnification; scale bar: 20 µm). Arrows indicate Gal^+^/ c-Fos^+^ neurons. Image of a Gal^+^/ c-Fos^+^ neuron is shown below (60X magnification; scale bar: 5 µm). **C**, **D** Pup retrieval induced c-Fos expression in the mPOA: **C** reduction in the fraction of c-Fos^+^/ Gal^+^ cells in MIA mothers (*t*_(9)_ = 2.769; *Ρ* = 0.022; *Ν* = 5–6 animals/group), but (**D**) no difference in the total number of Gal^+^ cells (normalized to DAPI, *N* = 5–6 animals/group). **E**, **F** Pup retrieval induced c-Fos expression in the vBNST: no difference in **E** the fraction of c-Fos^+^/ Gal^+^ cells (*N* = 5–6 animals/group) or (**F**) the total number of Gal^+^ cells between MIA and control dams (normalized to DAPI, *N* = 5–6 animals/group). c-Fos staining was analyzed in 1 image per hemisphere acquired from two sections per brain. **G**, **H** Single-cell RNA sequencing identifies Gal^+^ clusters in the MPN that co-express genes relevant for Poly I:C signaling: **G** sex-specific expressional profile of Poly I:C related genes in the Gal^+^ clusters i8, i16, and i18; 0: females; 1: males [34]. **H** Representative section of the mPOA schematically depicting the distribution of Gal^+^ clusters i8, i16 and i18 in the MPN (according to Fig. 7C [34]) using MERFISH and single-cell RNA sequencing. All data are presented as mean ± SEM, *Ρ  <  0.05.

We took advantage of *Gal::Cre//FlexEYFP* transgenic mice to directly visualize the Gal^+^ neurons in the mPOA upon MIA (Fig. 3A). On this background, we used c-Fos, an immediate early gene, to mark any rapid neuronal activation in response to pup-evoked care behavior [20] (Fig. 3B). When analyzing MIA dams 90 min after pup retrieval we found significantly lesser c-Fos immunoreactivity in Gal^+^ neurons. Residual c-Fos responses were particularly confined to Gal^+^ neurons in the medial preoptic nucleus (MPN), a mPOA subdivision specifically associated with parental behavior [34] (Fig. 3B, C). Notably, the absolute number of Gal^+^ neurons was unchanged between the MIA and control groups (Fig. 3D), substantiating that MIA might modulate their circuit involvement and functionality rather than survival per se.

Gal^+^ neurons are distributed along multiple nuclei of the rostral hypothalamus and associated basal forebrain nuclei that extend in the anterior direction. One such area is the bed nucleus of stria terminals with its ventral domain (vBNST) harboring a significant Gal^+^ neuronal contingent [34]. Considering the interplay of the vBNST with the mPOA in shaping parenting behaviors [50, 51], we asked if Gal^+^ neurons of the vBNST are also affected by gestational Poly I:C application. Neither c-Fos expression in Gal^+^ neurons nor the total number of Gal^+^ neurons were affected (Fig. 3B, E, F). These data suggest that reduced responsiveness of Gal^+^ neurons at the level of c-Fos expression to pup retrieval after MIA challenge might be a selective feature of those situated in the mPOA.

### Molecular profiling of Gal^+^ neurons in the mPOA

Galanin expression in the mPOA labels a substantial set of neurons, for which single-cell RNA-seq suggests the presence of phenotypically distinct subclasses. Exploring which subset contains genes pivotal for Poly I:C recognition and signaling together with their fast neurotransmitter contents seem imperative to specify the cellular sensitivity of the mPOA to Poly I:C. Moreover, this would allow to delineate both efferent projections/postsynaptic targets and amenable intersectional genetic strategies to build causality towards the neuronal basis of maternal care behaviors adversely affected by MIA. To this end, we have reprocessed the open-label database by Moffitt et al. [34], containing both single-cell RNA-seq and positional information on MPN neurons. Considering anatomical evidence for inhibitory mPOA-to-VTA afferents, the mPOA output thought to drive motivational aspects of parenting [52], we focused on Gal^+^ clusters expressing the GABAergic markers *Gad1*and *Slc32a1*, originally termed i8, i18 and i16, to search for candidate genes involved in Poly I:C signaling (see “Materials and methods”, Supplementary Fig. 3). In brief, Toll-like receptor 3 (TLR3) is central to the cellular recognition of Poly I:C in both immune and non-immune cellular lineages, the latter also including neurons [53]. TLR3 activation induces the production and release of inflammatory cytokines, in particular type I interferons (IFNs) [54]. We found that some Gal^+^ neurons in clusters i8 and i18 express *tlr3*, and that *tlr3* expression predominates in females. Coincidently, neurons in cluster i18 also express substantial levels of *Ifnar1*. Likewise, cluster i16 also expresses *Ifnar1* and *Ifnar2* (with higher levels in females over males), as well as Janus kinases (*Jak*) and other components of the interferon signaling cascade [55] (Fig. 3G, H). In sum, the transcriptional profile of Gal^+^ neurons in the MPN suggests a molecular framework for their preferential sensitivity to Poly I:C, either directly or through interferons released locally. Moreover, the GABA identity of Gal^+^ neurons allows for the hypothesis that impairments of their projection towards the VTA, inhibition of which disrupts pup retrieval [56], might be central to the behavioral pathobiology of MIA.

### Circuit specificity of Gal^+^ neurons in the mPOA that project to the VTA

**Fig. 4:**
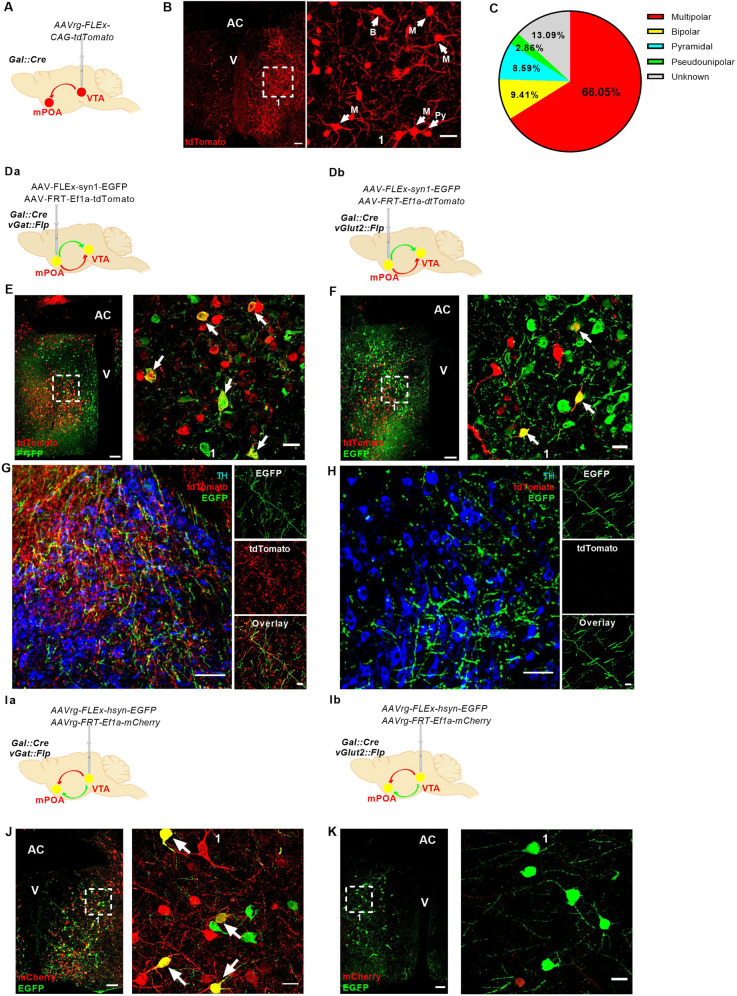
mPOA Gal^+^ neurons projecting to the VTA (mPOA Gal^+^ → VTA) are inhibitory and show mainly multipolar morphology. **A** Schematic representation of the viral strategy used for the retrograde labeling of mPOA Gal^+^ → VTA neurons. **B** Representative section of the mPOA with labeling of the mPOA Gal^+^ → VTA neurons (4 × 3 stitched 20X image; scale bar: 100 µm; AC anterior commissure, V ventricle). The white dashed box indicates the magnified area shown in (1) with representative immunostaining of morphologically distinct mPOA Gal^+^ → VTA neurons (20X magnification; scale bar: 40 µm; M multipolar, B bipolar, Py pyramidal). **C** Pie chart depicting the proportion of distinct morphological classes of Gal^+^ neurons in the mPOA (*Ν* = 3 animals, 2 sections/animal). **D** Schematic representations of the viral strategy used for the anterograde labeling of **Da** mPOA Gal^+^ and vGAT^+^ → VTA neurons and **Db** mPOA Gal^+^ and vGlut2^+^ → VTA neurons. **E**, **F** Representative section of the mPOA of **E** Gal::Cre/vGat::Flp and **F** Gal::Cre/vGlut2::Flp female mice injected with AAV-FLEx-syn1-EGFP + AAV-FRT-Ef1a-tdTomato (2 × 3 stitched 20X image; scale bar 100 µm; AC: anterior commissure; V: ventricle). The dashed square indicates the magnified area illustrated in 1. White arrows mark **E** Gal^+^/vGat^+^ and **F** Gal^+^/vGlut2^+^ neurons (60X magnification; scale bar: 20 µm). **G**, **H** Representative sections of the VTA of **G** Gal::Cre//vGat::Flp and **H** Gal::Cre//vGlut2::Flp females after injection of AAV-FLEx-syn1-EGFP and AAV-FRT-Ef1a-tdTomato in the mPOA (×20 magnification; scare bar: 20 µm). Images of exemplary immunostained axons are shown on the right (60X magnification; scale bar: 10 µm; four sections from two animals per genotype were considered for qualitative evaluation). **I** Schematic representations of the viral strategy used for the retrograde labeling of **Ia** mPOA Gal^+^ and vGAT^+^ → VTA neurons and **Ib** mPOA Gal^+^ and vGlut2^+^ → VTA neurons. **J**, **K** Representative section of the mPOA of **J** Gal::Cre/vGat::Flp and **K** Gal::Cre/vGlut2::Flp female mice injected with AAVrg-FLEx-hsyn-EGFP + AAVrg-FRT-Ef1a-mCherry (2 × 3 stitched 20X image; scale bar 100 µm; AC: anterior commissure; V: ventricle). The dashed square indicates the magnified area illustrated in 1. White arrows mark Gal^+^/vGat^+^ neurons (60X magnification; scale bar: 20 µm; two sections from one female per genotype were considered for qualitative evaluation).

Even though Gal^+^ projections from the mPOA to the VTA (mPOA → VTA) were implicated in the control of motivational aspects of maternal behavior [25], the characterization of these monosynaptic mPOA efferents remains incomplete. Here, we first injected AAVrg-FLEx-CAG-tdTomato viruses into the VTA of *Gal::Cre* nulliparous females to retrogradely map the extent of mPOA → VTA and specify their morphological properties (Fig. 4A and Supplementary Fig. 4). tdTomato signal was abundant in the mPOA, where we identified 4 morphologically distinct neuronal subpopulations. Amongst these Gal^+^ neurons, 65.5 ± 4.3% were multipolar and therefore morphologically reminiscent of the Golgi–Cox-stained neurons affected by MIA (Fig. 4B, C).

Considering that more than 80% of Gal^+^ neurons also express estrogen receptor 1 (Esr1) [57] and that E*sr1*^+^ mPOA → VTA projections are inhibitory [52], we tested the hypothesis that mPOA Gal^+^ → VTA neurons co-express GABA by an intersectional genetics approach. We injected a cocktail of anterograde AAVs (AAV-FLEx-syn1-EGFP + AAV-FRT-Ef1a-tdTomato) in the mPOA of Gal::Cre//Vgat::Flp and Gal::Cre//Vglut2::Flp mice to visualize Gal in green and *Vgat* or *Vglut2* in red, respectively. (Fig. 4Da, Db). As predicted by single-cell RNA-seq [34], Gal^+^ neurons in the mPOA can be both excitatory and inhibitory, marked by vesicular glutamate transporter 2 (VGLUT2) and vesicular GABA transporter (VGAT), respectively (Fig. 4E, F). However, Gal^+^ axons exclusively co-expressing VGAT were found in the VTA, identifying the mPOA → VTA projection as inhibitory (Fig. 4G, H). To confirm these results, we have also performed retrograde tracing in both Gal::Cre//Vgat::Flp and Gal::Cre//Vglut2::Flp mice by co-injecting AAVrg-FLEx-hsyn-EGFP (to visualize Gal in green) and AAVrg-FRT-Ef1a-mCherry (to label either GABAergic or glutamatergic cells in red) viruses (Fig. 4Ia, Ib). Many Gal^+^ neurons in the mPOA of Gal::Cre//Vgat::Flp were dual-labeled, while no such neuron was found in Gal::Cre//Vglut2::Flp mice (Fig. 4J, K). These are qualitative observations describing the neurochemical characteristics of the mPOA Gal^+^ → VTA pathway and no quantifications and statistical evaluations have been conducted for these data sets.

Dendritic spines are generally considered as the postsynaptic targets of excitatory inputs, including in the VTA [58], and their shape and size are taken as a measure of synaptic plasticity and remodeling [59]. Therefore, we assumed that the impact of MIA on GABAergic mPOA → VTA afferents shall not affect either the density or shape of dendritic spines in the VTA unless MIA independently impacts the VTA circuitry. Analysis of dendritic spines on type I and type II neurons [60] in the VTA (Supplementary Fig. 5A) revealed no effect of immune activation on spine density in either mothers or nulliparous females (Supplementary Fig. 5B). We then classified dendritic spines into filopodia, mushrooms, stubby, thin and long thin as per standard terminology [27], yet did not find alterations of any spine type after MIA (Supplementary Fig. 5C–G).

Cumulatively, these data show that the Gal^+^ multipolar neuron population that is sensitive to MIA projects to the VTA and suggest that the dampened activity of an inhibitory mPOA → VTA projection in MIA brains could pathologically shift neuronal activity within the VTA.

### Altered single-unit activity in VTA coincides with impaired pup retrieval upon MIA

**Fig. 5:**
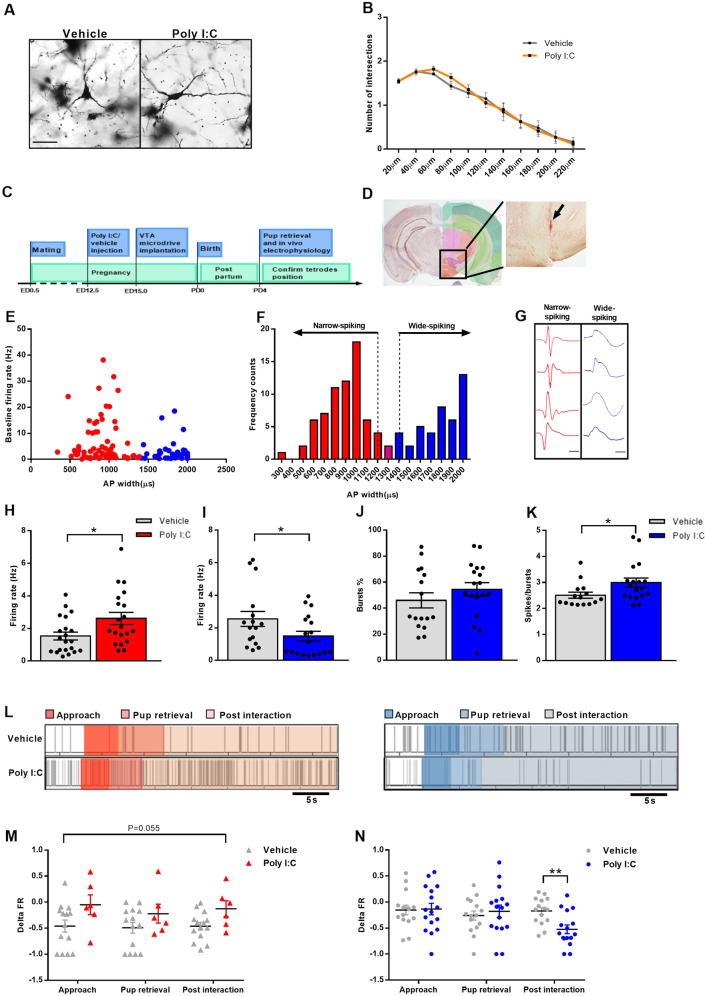
VTA single units show altered firing rate (FR) during baseline and pup exposure. **A** Examples of Golgi–Cox impregnated VTA neurons from vehicle and Poly I:C-injected mothers (20X magnification; scale bar: 50 μm). **B** Poly I:C injection does not result in alterations of number of intersections in VTA neurons of mothers (*N* = 5 animals/group). The morphology of six neurons was reconstructed from two sections per animal for a total of 30 neurons per group. **C** Schematic depiction of the experimental timeline for in vivo electrophysiology. **D** Representative coronal section from a control mother showing the tetrode tract mark in the VTA region (arrow) (adapted from Allen Brain Atlas; Bregma: −2.555). **E** Baseline FR versus full AP widths of recorded single units (*N* = 111 neurons; five animals/group; red: narrow-spiking neurons; blue: wide-spiking neurons). **F** Frequency histogram of full AP widths showing a bimodal distribution. Cutoffs of 1200 µs and 1400 µs spike width were used to differentiate between two subpopulations of neurons (narrow-(red) and wide-(blue) spiking neurons) within the VTA (arrows). Single units with spike width comprised between 1200 µs and 1400 µs were assigned to either group based on defined waveform characteristics or excluded from the analysis (*N* = 111 neurons; 5 animals/group). **G** Examples of single units’ waveforms from narrow-(red) and wide-(blue) spiking neurons (scale bar: 500 µs). **H**, **I** Poly I:C injection during pregnancy leads to **H** significant FR increase of narrow-spiking neurons (*U* = 122; *Ρ* = 0.022; *Ν* = 20–21 neurons/group, five animals/group) and **I** significant FR reduction of wide-spiking neurons (*U* = 86; *Ρ* = 0.046; *Ν* = 16–18 neurons/group, five animals/group). **J**, **K** Poly I:C-injected mothers **J** do not show any change in bursts percentage (%) of wide-spiking neurons (*N* = 16–18 neurons/group; five animals/group) but (**K**) have significantly altered number of spikes/bursts (*U* = 75.5; *Ρ* = 0.03; *Ν* = 16–18 neurons/group; five animals/group). *Ρ <  0.05. **L** Sample raster plots of narrow- and wide-spiking neurons during retrieval of the last pup in MIA and control mothers; behavioral epochs are marked in different shades of colors (from left to right: approach, pup retrieval and post interaction. Narrow-spiking neurons: shades of red; wide-spiking neurons: shades of blue). **M** Narrow-spiking neurons show a trend for alteration in FR change during the three behavioral epochs in Poly I:C-injected mothers. Ρ value indicates the main effect of treatment (main effect of treatment: *F*_([1)_ = 4.230; *Ρ* = 0.055; *Ν* = 6–14 neurons/group; 3–4 animals/group). **N** Wide-spiking neurons show altered FR change after Poly I:C injection during the post interaction behavioral epoch. Asterisks refer to the pairwise comparison. Delta FR: calculated according to the standardized change ratio described in “Materials and methods” (interaction of treatment and behavior: *F*_(2)_ = 4.954; *Ρ* = 0.01, *Ν* = 15–16 neurons/group; 3–4 animals/group). All data are presented as mean ± SEM, *Ρ < 0.05, **Ρ < 0.01.

Neuronal reconstruction and subsequent Sholl analysis did not reveal differences in any of the dendritic parameters analyzed in MIA dams relative to controls (Supplementary Fig. 5H, I and Fig. 5A, B). However, dysfunctional afferent inputs from the mPOA could bias the activity of VTA neurons. Therefore, we investigated the electrical activity of VTA neurons in MIA and control dams in the context of maternal behavior. Single-unit activity was recorded for 10 min under baseline conditions (without pups) and an additional 15 min of pup retrieval (Supplementary Videos 3 and 4; see timeline in Fig. 5C). Electrode placement was confirmed in postmortem brain sections upon Cresyl violet staining (Fig. 5D). Based on firing rates (FRs) and the frequency distribution of pooled spike widths, we were able to classify VTA neurons into three subpopulations: fast-firing neurons (FR > 10 Hz) and two populations of neurons with low FR (FR < 10 Hz): narrow-spiking neurons and wide-spiking neurons (Fig. 5E, F).

Fast-firing neurons were only detected in some of the animals (five out of ten). We therefore, focused on narrow- and wide-spiking low FR neurons. Most narrow-spiking, putative GABAergic neurons had very short action potential (AP) duration [61, 62]. In contrast, wide-spiking neurons showed typical features of dopaminergic neurons including long AP duration, slow depolarization and large negative undershoot [61–66] (Fig. 5G). Narrow-spiking putative GABAergic neurons from MIA mothers had significantly higher FR compared to controls (Fig. 5H). In contrast, FR from wide-spiking neurons (putative dopaminergic neurons) was significantly lower in MIA females than controls (Fig. 5I). Dopaminergic neurons can fire either in a tonic mode or in a burst pattern [33]: tonic firing contributes to novelty-gated information storage, while burst firing is involved in reward [67]. Bursting percentage (%) was not altered by MIA in wide-spiking neurons (Fig. 5J). However, the number of spikes per burst was significantly higher in gestational Poly I:C-injected mice than controls (Fig. 5K). This bidirectional regulation (that is, hyperactivity of putative GABAergic vs. hypoactivity of putative dopaminergic cells) suggests a possible interplay between the two subpopulations of VTA neurons and the adverse effect of MIA thereon.

Next, we determined spiking activity during different behavioral epochs in the pup retrieval assay, including approach, pup retrieval and post interaction (Fig. 5L). Individual FRs were normalized to baseline average FR and individual change indices (ΔFR) were calculated for each behavioral epoch and compared across groups. Consistent with baseline results, narrow-spiking neurons had lower ΔFR (that is, higher absolute FR), in MIA dams as compared to controls (Fig. 5M). Analysis of wide-spiking neurons revealed that MIA and control mothers significantly differed in the last behavioral epoch with wide*-*spiking neurons in MIA dams having significantly higher ΔFR (conferring lower absolute FR; Fig. 5N).

**Fig. 6:**
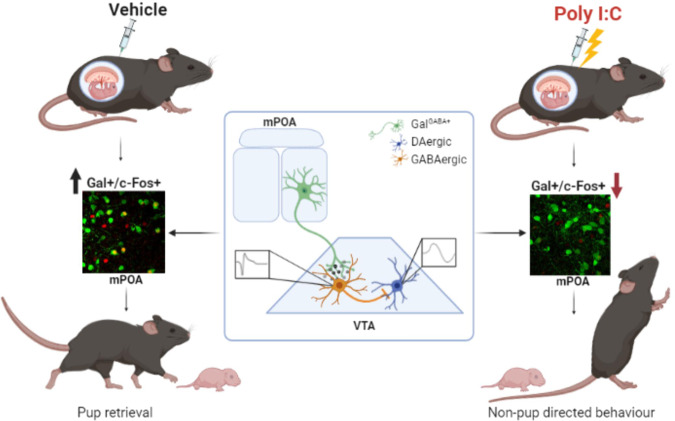
Proposed model. Reduced activation of mPOA Gal^+^ neurons upon pup retrieval in dams after gestational Poly I:C treatment. mPOA Gal^+^ → VTA projections are inhibitory; reduced activation of mPOA Gal^+^ in MIA dams could result in disinhibition of trigger GABAergic neurons in the VTA and subsequent bias in the engagement of non-pup-related over pup-related (retrieval) behavior (made with Biorender.com).

Collectively, these data suggest that reduced activation of mPOA Gal^+^ neurons during pup retrieval leads to disinhibition of putative GABA neurons in the VTA, leading to a decreased excitation of putative dopamine neurons. These changes may account for a reduction of reward associated with retrieving the pups back to the nest, biasing MIA mothers to engage in non-pup-directed exploratory behavior at the expense of protecting their offspring (Fig. 6).

## Discussion

The long-term consequences of gestational infection in the MIA model of viral-like immune activation on offspring brain development and behavior have been comprehensively studied and strongly related to severe psychopathologies, including autism spectrum disorder, schizophrenia and major depression [13, 68, 69]. In contrast, much less attention has so far been directed towards investigating the sequelae of MIA on maternal behavior and the maternal brain itself although cross-fostering studies have highlighted that both prenatal and postnatal factors contribute, albeit distinctively, to behavioral and neuroanatomical/ -chemical abnormalities in the offspring [8, 9, 70, 71].

Here, we used the Poly I:C model of viral infection to dissect out that MIA rewires the maternal brain such that care behavior becomes impaired due to structural modifications of mPOA neurons, amongst which multipolar neurons (including Gal^+^ to VTA projection GABA^+^ neurons) are most affected. Even though brain-wide tracing of afferent and efferent connections to and from the mPOA was not performed here, we could show a disruptive effect of MIA on the activation of mPOA Gal^+^ neurons and reduced excitatory drive from dopaminergic output neurons of the VTA in MIA dams. This observation is important since VTA, a part of the core reward circuitry [72] with major corticolimbic projections to the prefrontal cortex and nucleus accumbens [73], drives motivational aspects of parenting behaviors [25]. This notion was reflected in disrupted pup-directed exploration and retrieval by MIA dams even if sensorial perception of pup cues by the mother and pup physiology remained intact. Instead, dams exposed to MIA preferentially engage in explorative rearing instead of protecting their offspring by retrieval, pinpointing their failure to properly identify possible environmental dangers for their pups. Thus, we see a robust and counterproductive behavioral phenotype that contrasts the evolutionary privilege of behaviors to ensure the species’ survival when retrieval becomes inferior to exploratory drive after MIA. In light of previous reports of cross-fostering studies that convincingly contrasted the differential impact of MIA-induced prenatal disruption of fetal brain development, versus alterations in postnatal maternal factors on offspring phenotypes, it remains to be explored how the herein observed impact of gestational Poly I:C treatment on the wiring of the maternal brain affects brain and behavioral function in the offspring [8, 9, 70, 71].

mPOA neurons are pivotal for parenting behavior [20]. Our study provides evidence for cytoarchitectonic modifications along their somatodendritic axis and their axonal projections. An open question is if MIA-induced reorganization of neuronal structure occurs acutely (e.g. by phasic cessation of gene regulatory networks supporting structural plasticity) or through long-lasting epigenetic mechanisms, particularly accessing chromatin accessibility or premature mRNA or protein degradation. The long-lasting nature of structural deficits invites considerations about epigenetic mechanisms, particularly because prenatal and early postnatal exposure to Poly I:C affect DNA methylation, posttranslational modifications of histones and miRNA expression [15, 16, 18, 74, 75]. Surprisingly limited evidence for epigenetic modifications of the mPOA in the context of parental behavior is available [76]. Nevertheless, and relying on other trans-generational studies in which histone modifications take primary roles in changing stereotyped behaviors [29, 77, 78], we posit that compromised chromatin accessibility could reduce structural plasticity of the maternal brain.

The finding that morphological alterations in the MIA mPOA compromise multipolar neurons is most relevant given that they comprise the largest Gal^+^ neuronal contingent [30, 79]. Thus, while it is integral to our hypothesis that these multipolar neurons and their VTA projections are affected by MIA, we also suggest that other subsets within this multipolar Gal^+^ neuron pool could exist whose equivalent reorganization could challenge other behaviors such as coping, reward-seeking, mating or even the maintenance of the diurnal cycle. Thus, making further molecular subdivisions within this Gal^+^ cluster, through improved next-generation sequencing strategies, could reveal additional layers of local circuit reorganization, which could pose a risk for mothers in behavioral contexts unrelated to parenting.

Indeed, we found through the analysis of mPOA single-cell RNA-sequencing data that *tlr3*, which recognizes double-stranded RNA, including Poly I:C [53], is expressed in Gal^+^ neurons and more strongly in females than in males, as are receptors of the cytokines induced by Poly I:C signaling, including *Ifnar1* and *Ifnar2*. However, not all Gal^+^ neurons expressed *tlr3*, *Ifnar1,* and *Ifnar2* mRNAs at the time point of sequencing of open-source data. There are a number of possibilities given this heterogeneity: (i) the expression profiles of these genes are environment-driven, or metabolism-driven and could fluctuate during pregnancy; (ii) a small subset of cells alike “hub” neurons in the hippocampus or neocortex [80, 81] could imprint large-scale neurocircuit modifications as starter cells. Thus, a circumspect number of expression foci could be sufficient for re-entraining the mPOA; (iii) neither single-cell RNA-seq nor epigenome-related sequencing data exist on glial cells of the mPOA, or more broadly the brain. Therefore, we can only hypothesize that microglia, which are relevant to behavioral alterations in MIA offspring [82, 83], could act as local entry points for Poly I:C. This concept is supported by the abundance of *tlr3* and expression of receptors of cytokines induced by Poly I:C, including *Ccr1/5/6, Cxcr2/4, Il6ra, Il10ra, Il17ra, tnfr1a/b* in mouse microglia [84]. Thus, the compromising insult could be indirect on inhibitory Gal^+^ neurons, which in the mPOA abundantly express 20 different cytokine and chemokine receptors (Supplementary Fig. 6). Alternatively, and considering that hypothalamic astrocytes change their gene expression profiles when recruited to functionally-specified neurons to provide metabolic support (RAR and TH, unpublished), we foresee a role for astroglial transformation in compromising the metabolic and structural integrity of multipolar Gal^+^ neurons.

The mPOA Gal^+^ → VTA circuitry is critical for maternal motivation under physiological conditions [25] but has not yet been related to dysfunctional maternal care behavior in disease models. This pathway is exclusively inhibitory with Gal^+^ neurons projecting onto GABAergic rather than dopaminergic VTA neurons. Our results support this network layout with reduced inhibition falling onto GABA neurons in the VTA and their subsequent disinhibition manifesting as reduced dopamine output [85]. However, if mPOA Gal^+^ cells actually release Gal in the context of parenting behavior and the role of Gal itself in the mPOA → VTA circuitry, maternal motivation and its disruption by MIA, is entirely elusive at this point. Moreover, it is conceivable that neural pathways other than mPOA Gal^+^ → VTA are contributing to the behavioral phenotypes of MIA mothers: (i) the observed alterations of GABAergic and dopaminergic activity in the VTA of MIA mothers may also be independent on the mPOA input. VTA neurons express cytokine receptors, including some relevant to Poly I:C signaling [86, 87] and their activation could directly impact on VTA neurons to modulate intrinsic properties of neuronal excitability; (ii) alternatively, other brain regions involved in maternal behavior could play a part, such as the medial prefrontal cortex (mPFC), which is highly interconnected to the VTA 16 and is also activated by maternal behavior in both rodents and humans [88–90]; (iii) reduced activation of mPOA Gal^+^ cells could also affect other output regions of the mPOA previously implicated in maternal behavior, including mPOA Gal^+^ → PAG and mPOA Gal^+^ → MeA, both also important for the execution of pup-directed behaviors [25]. The hyperactivity of GABAergic and reduced activity of VTA dopaminergic neurons during impaired pup retrieval in MIA dams similar to what is shown here, are prominently related to the action of ethanol, cannabis, cocaine and other drugs of abuse and their modulation by stressful or aversive stimuli [66, 91, 92]. The resulting reduction in dopaminergic drive and corresponding decrease in the experience of reward could substantially bias maternal decision-making to disfavor protection and offspring caretaking over other instinctive, non-pup-directed activities, such as exploration of a novel environment. Yet, such changes are unlikely to be sex-specific because mPOA → VTA projections are also relevant to parenting behavior in males [25]. Thus, future studies could disentangle if paternal behaviors are equally compromised by viral (-like)viral infections.

These notions are significant not least against the background of the current COVID 19 pandemic, leaving not only hundreds of thousands of women worldwide with a viral infection during pregnancy (270,000 in the Americas alone, as of September 2021 [93], but also both sexes with the debilitating outcomes of “long COVID”, including an increased incidence of mood, anxiety and substance abuse disorders [94]. Therefore, the line of research which our paper heralds, is of direct public health relevance.

In conclusion, our findings propose that gestational immune activation leads to long-lasting alterations in the maternal brain, which reduce the motivation to nurture and protect the infant. From a translational perspective, this suggests that infection during pregnancy may disrupt mother-infant-interaction postpartum herby not only impairing maternal health and well-being, but also endangering the physical, cognitive, and emotional development of the child.

## Supplementary information

Supplementary Materials and Methods

Supplementary Tables

Supplementary Figure Legends

Supplementary Figure 1

Supplementary Figure 2

Supplementary Figure 3

Supplementary Figure 4

Supplementary Figure 5

Supplementary Figure 6

## Data Availability

scRNA-seq data were reprocessed from Moffitt et al. [34] and are available on Gene Expression Omnibus (GEO) (GSE113576).
