# Supplementary material for: Gestational immune activation disrupts hypothalamic neurocircuits of maternal care behavior
Source: Mol Psychiatry. 2022 May 17;29(4):859–73. doi: 10.1038/s41380-022-01602-x (PMC9112243; doi:10.1038/s41380-022-01602-x)
Supplement: Supplementary file 1 — Supplementary Materials and Methods [file 41380_2022_1602_MOESM1_ESM.docx]

# **Supplementary materials and methods**

1. **Animals**

Animal experiments were done following the ARRIVE ethical guidelines and the U.K. Animals Scientific Procedures Act, 1986 and associated guidelines (EU Directive 2010/63/EU for animal experiments). The national ethical committee of animal care and use approved all animal procedures and experiments (2020-0.193.053 and AC-AAAS1500 for in-vivo electrophysiology).

Animals were housed in light, temperature, humidity, and CO_2_ concentration standard conditions with a 12-hour light-dark cycle (light phase between 8am and 8pm) and 21-23°C ambient temperature. Food and water were provided ad libitum.

2-5 months old, C57Bl/6N female mice were used for all WT experiments including MIA, behavioral studies, Golgi-COX staining and single unit in vivo electrophysiology.

Transgenic lines included *Gal::Cre* mice, *FlexEYFP* females, *vGat::Flp* and *vGlut::Flp* mice (**Supplementary Table 2**). 2-4 months old, C57Bl/6J Gal::Cre transgenic female mice (B6.FVB(Cg)-Tg(Gal-cre)KI87Gsat/Mmucd, stock number 036969-UCD) were obtained breeding *Gal::Cre* males and WT females and kept in a hemizygous status according to the protocol provided by the Mutant Mouse resources and research centers (MMRC). *Gal::Cre* males were bred with homozygous FlexEYFP females (JAX stock #006148, Jackson Laboratories, Bar Harbour, ME, USA) to obtain Gal::Cre//FlexEYFP female mice. *Gal::Cre* and *Gal::Cre//FlexEYFP* female mice were used respectively for viral procedures and c-Fos staining upon MIA. *vGat::Flp//Gal::Cre* and *vGlut::Flp//Gal::Cre* female mice were obtained crossing *Gal::Cre* females and respectively *vGat::Flp* and *vGlut::Flp* males (respectively JAX stock #029591 and #030212, Jackson Laboratories, Bar Harbour, ME, USA) and were used for anatomical characterization.

1. **Maternal immune activation**

A timed mating procedure was applied for all MIA experiments as previously described (1). 2-3 months old females were group housed for at least 1 week for synchronizing the oestrous cycle according to the Lee-Boot effect (2) and subsequently exposed to 72 hours bedding switch in order to make them receptive for mating (Whitten effect (3)). The day after the mating was considered as ED0.5 and the day of birth as PD0 in all instances. Mothers were monitored for weight gain at ED7.0, ED12.5 and ED18.5. Pups were counted and weighted at PD0 and PD4. Accesses to water and food was recorded daily in two sessions from PD1 to PD6 (4,5) between 11am and 1pm and between 3pm and 5pm with cameras (Axis communications, Lund, Sweden). Videos were scored every three minutes by Observer software (Noldus, Wageningen, the Netherlands).

Poly I:C was obtained from Sigma (P9582, Vienna, Austria), dissolved in 0.9 % NaCl solution (vehicle) and injected at ED12.5 at a concentration of 20mg/Kg according to the standard protocol used in our laboratory (5–7). Pregnant females received either 10uL/g of Poly I:C or 0.9% NaCl solution i.p.. All experiments were conducted following the standard conditions recommended by Kentner and colleagues (8) (**Supplementary** **Table 3**). Sickness behavior was evaluated 2 hours after Poly I:C versus vehicle injection in pregnant mice. Mice were scored for the presence of 4 different parameters including body posture, ptosis, piloerection and nest condition. The severity of sickness behavior was assessed using a four levels score (0-3) for each parameter, where 0 corresponded to no sign of sickness behavior (own protocol adapted from (9,10); **Supplementary** **Table 4**). ). The presence or absence of diarrhea was also evaluated. Body weight of all mice was determined 24 hours after immune activation. Mice that did not exhibit both weight loss and apparent sign of sickness behavior, were not considered for the analysis.

1. **Behavioral experiments**

All behavioral experiments were conducted during the light phase of the light/ dark cycle and light and temperature conditions were maintained constant at all times. Animals were allowed to habituate to the experimental room for at least 1 h before testing.

***Pup retrieval assay***

The pup retrieval assay was conducted at PD4 under dim light conditions (19-20 Lux) following a standard procedure (11). Briefly, the mother was separated from the pups for 20 minutes before 4 randomly selected pups were returned back into the home cage on the opposite side of the nest. The mother was placed at the center of the cage and behavior was recorded for 5 minutes using a commercially available Webcam (Logitech, Lausanne, CH) and the Media Recorder software. The time required to retrieve all pups back to the nest was analysed by EthoVision XT software (Noldus, Wageningen, the Netherlands). Mothers, who failed to retrieve the pups, were given the maximum time of the recorded session (300s). To exclude any effect in behavior due to litter size, only dams having 6-10 pups per litter were considered for analysis.

***Ultrasonic vocalizations analysis (USVs)***

USVs were recorded and analysed at PD4. 4 pups were randomly selected from each litter and tested individually for the presence of USVs inside a noise insulated box (Noldus, Wageningen, the Netherlands), equipped with an USB Ultrasound microphone (from Petterson, part of the Ultravox system) and recorded for 3 minutes (Gain=95). The sonograms were analysed using the UltraVox XT software (Noldus, Wageningen, the Netherlands) and parameters considered were duration of calls, amplitude (represented as linear amplitude), frequency at maximum peak and total number of calls per litter. Calls were further divided into different categories (*Short, Flat, Chevron, Complex, Upward, Downward, Two-syllables and Three-syllables*) and analysed based on adapted previous published protocol (12) (**Supplementary** **Table 1**).

1. **Golgi-Cox staining**

The FD Rapid GolgiStainTM Kit (FD Neurotechnologies, inc, Maryland, United States) was used for the Golgi-Cox staining procedure for neuronal reconstruction of postpartum (PD7) and nulliparous control MIA and vehicle treated females following the manufacturer’s instructions. After cervical dislocation the brain was extracted and merged in an equal combination of solution A and solution B for 12 days and in solution C for 3-7 days. The brain was frozen in isopentane: briefly, a metal beaker was filled with isopentane, placed in liquid nitrogen and the brain dipped in isopentane for 20-30 seconds and subsequently in dry ice for 5 minutes. 150µm slices were cut using the cryostat at -22 °C at the level of the mPOA (Bregma +0.26 and -0.22) and the VTA (bregma -2.92 and -3.88) regions (13). Slices were further processed following the protocol provided by the manufacturer. Slices were also stained with 0.1% Cresyl violet solution (Merck, Darmstadt, Germany) for 7 minutes.

***Neuronal reconstruction and morphological analysis***

Neurolucida 10 (MBF Bioscience, Vermont, United States) was used for reconstruction of the neuronal morphology in the mPOA and in the VTA of Golgi-Cox stained brains. Reconstructions were conducted by an experimenter blinded to the experimental groups. Neurons considered for reconstruction had the cell soma fully impregnated, distinguishable from neighbouring processes and found in the middle of the slice to avoid cutting of projections. Since axons are often not impregnated or short, they were excluded from the reconstruction. In the mPOA two 300µm x 300µm digitized boxes were selected in both hemispheres above the region of interest, using as dorsal and medial boundaries the anterior commissure and the third ventricle and only neurons with cell bodies present within this box were considered for analysis (14). Three different subpopulations of neurons were identified based on soma shape and number of dendrites: bipolar neurons (two dendrites projecting to opposite directions and fusiform/round cell body), pyramidal neurons (three dendrites organized in a pyramidal shape and pyramidal cell body) and multipolar neurons (3-6 dendrites spreading in all directions and round/fusiform cell body) (15). Neuronal morphology was analysed in N = 6 animals/group: 4-5 multipolar neurons were drawn in two 150µm sections per animal (Bregma +0.14 and 0.02), with a total of 24-30 neurons per group. Parameters analysed were cumulative length of dendritic tree, number of nodes and area of cell soma. A Sholl analysis was conducted to calculate the number of dendritic intersections along concentric circles with radius (r) = 20µm; r1 = r + 20 µm; r2 = r1 + 20 µm etc. and maximum radius being r= 220 µm.

A digitized box was drawn above the VTA of both hemispheres (Bregma -2.92 and -3.28) considering as medial and lateral boundaries the fornix and the medial lemniscus. The morphology of 6 neurons was reconstructed from two 150µm sections per animal for a total of 30 neurons per group (N = 5 animals/group).

The density of spines was determined on primary/ secondary dendrites at segments with at least 5-10 µm distance from cell bodies, dendritic ramifications, and terminals (total 655-700 µm in length in each mouse). Spines shorter than 0.21µm were not considered for analysis. Spines were also classified into *stubby, mushroom, thin, long thin and filopodia* based on the algorithm provided by (16). The number of spines was counted in four segments of VTA primary dendrites and VTA secondary dendrites from 2/3 neurons per brain hemisphere (N = 16 segments/mouse).

1. **Viral tracing**

***Viruses***

Information on all viruses used is summarized in **Supplementary Table 2**. The anterograde adenoviruses (AAVs) AAV-FLEx-syn1-EGFP and AAV-FRT-Ef1a-tdTomato were stereotactically injected into the mPOA of *vGat::FlpO//Gal::Cre* and *vGlut::FlpO//Gal::Cre* females to enable the expression of EGFP and tdTomato in galanin and respectively vGAT/ vGLUT2-expressing neurons.

The retrograde adenoviruses (AAVrg) AAVrg-FLEx-CAG-tdTomato, AAVrg-FLEx-hsyn-EGFP and AAVrg-FRT-mCherry were stereotactically injected into the VTA of *Gal::Cre*, *vGat::FlpO//Gal::Cre* and *vGlut::FlpO//Gal::Cre* females to allow for the tracking of the inputs from the mPOA to the VTA by means of either tdTomato, EGFP or mCherry expression.

AAV-FLEx-syn1-EGFP (1:5 diluted) and AAV-FRT-Ef1a-tdTomato (undiluted) were mixed and 300nL were injected in the left hemisphere of the mPOA of *vGat::FlpO//Gal::Cre* (1:1) and *vGlut::FlpO//Gal::Cre* (1:3) females using a 30° angle to avoid damage of the sagittal sinus and resistance due to the lateral ventricles and anterior commissure (coordinates: AP= +0.3, ML= -2.6, DV= -5.0).

200 nL of AAVrg-FLEx-CAG-tdTomato (undiluted) was injected in the left hemisphere of the VTA of *Gal::Cre* females (coordinates: AP= -3.0, ML= -0.5, DV= -4.6), while AAVrg-FLEx-hsyn-EGFP and AAVrg-FRT-Ef1a-mCherry were mixed and 300nL were injected in the left hemisphere of the VTA of *vGat::FlpO//Gal::Cre* (1:1) and *vGlut::FlpO//Gal::Cre* (1:3) females (coordinates: AP= -3.0, ML= -0.5, DV= -4.6).

***Stereotactic surgery for virus injection***

mPOA coordinates were selected based upon Mouse Brain Atlas (13) and on previous published literature (11,17). Deep anaesthesia was induced by placing the mouse inside an induction chamber with oxygen (medical oxygen, Air liquid Austria GmbH, Schwechat, Austria) flow rate of 900 mL per minute and 4% Isoflurane concentration (Forane, Baxter, Deerfield, IL, USA). Isoflurane was administered by a Combi-vet® aneasthesia system (Rothacher Medical, Berne, Switzerland) for 1 minute: deep anaesthesia was confirmed by the absence of the toe withdrawal reflex. The head of the mouse was fixed in the stereotactic frame (Model 1900, David Kopf Instruments, Tujunga, CA, USA) and constant anaesthesia was delivered at an oxygen flow rate of 900 mL per minute and 2-2.5% Isoflurane concentration (Forane, Baxter, Deerfield, IL, USA). An incision was made along the sagittal suture to expose the skull for the identification of Bregma and Lambda using a centring scope 40x (Model 1915, David Kopf Instruments) and the brain aligned using an alignment indicator (Model 1905, David Kopf Instruments). AAVs were injected at given coordinates with constant rate of 50ηL per minute, using a micropump (TJ-1A-Micro Flow Rate Syringe Pump, Longer, Hebei, China) provided of a 10 µL Hamilton syringe (Model 701 RN SYR, Small Removable NDL, 32 ga, 2 in, point style 3, Hamilton Company, Reno, USA; distributor: Sigma, Vienna, Austria). Postoperative analgesia consisted in one single i.p. injection of Tramadolhydrochlorid (Tramal®, 100 mg, Grünenthal Gmbh, Germany) at 60 mg/kg and 2 days of oral administration (via drinking water) of Carprofen (Rimadyl®, 50mg/mL, Zoetis Österreich GmbH, Wien, Austria) 7.5 mg/mL. Viral expression was evaluated 3-4 weeks after injection of AAVs.

1. **Phospho-c-Fos (c-Fos) analysis**

For c-Fos analysis *Gal::Cre//FlexEYFP* females were sacrificed 90 minutes after the pup retrieval test and perfused according to the procedure described below.

***Perfusion and brain processing***

Mice were perfused with 20mL Phosphate Buffer Solution (PBS) 1x and 20mL Paraformaldehyde (PFA) solution 4%. After extraction, brains were fixed in PFA 4% solution overnight at 4 °C and kept in sucrose solution 30% (in DH_2_O) for 3 days at 4 °C. They were then embedded in Optimal Cutting Temperature (O.C.T.) (Tissue Plus, O.C.T. Compound, Scigen Scientific Gardena, CA, USA) medium, frozen in liquid nitrogen and stored at -80 °C until further use.

***Fluorescent immunohistochemistry***

Brains were cryosectioned in 30µm coronal slices and stored at -20 °C in cryoprotectant solution (30% glycerol, 30% ethylene glycol and 40% PBS 1x) until further use.

For c-Fos analysis in *Gal::Cre//FlexEYFP* female mice, slices containing the mPOA region (between Bregma +0.02 and Bregma -0.1) were selected for immunostaining. They were rinsed 3 times 20 minutes each in PBS 1x, incubated in Triton X-100 (Sigma-Aldrich, Darmstadt, Germany) 0.3% for 5 minutes to allow the permeabilization of the tissue and then in blocking solution (Triton X 0.1% and goat Serum 5% (DAKO, Glostrup, Denmark) in PBS 1x for 1 hour. Slices were incubated in a mixture of primary antibodies diluted in the blocking solution for 48 hours at 4 °C. For the mPOA, antibodies used were anti Phospho-c-Fos (D82C12, Cell Signalling Technology Inc., Frankfurt am Main, Germany) 1:8 000 and anti GFP (ab13970, Abcam plc, Cambridge, UK) 1:800. After washing in PBS 1x (as described above), slices were then incubated in a mixture of secondary antibodies in a solution made of goat serum 1% and Triton X 0.1% in PBS 1x. Secondary antibodies included Alexa Fluor™ 647-conjugated goat anti rabbit 1:600 (A-21244, Invitrogen, Life Technologies Corporation, Eugene, OR, USA) and Alexa Fluor 488-conjugated goat anti chicken 1:500 (A-11039, Invitrogen, Life Technologies Corporation, Eugene, OR, USA). Slices were rinsed, incubated in DAPI 1:1 000 (D9542, Merck, Sigma-Aldrich, Darmstadt, Germany) 5 minutes and finally mounted with fluorescence mounting medium (DAKO, Carpinteria, CA, USA).

For tracing and morphological experiments in *Gal::Cre*, *Gal::Cre//FlexEYFP*, *vGat::FlpO//Gal::Cre* and *vGlut::FlpO//Gal::Cre* females, sections of the mPOA and VTA regions (Bregma +0.02 and -0.1, Bregma -2.92 and Bregma -3.16) were immunostained, following the procedure described above. Primary antibodies used were anti-GFP (ab290, Abcam, Cambridge, UK) 1:800, anti-mCherry (CPCA-mCherry, EnCor Biotechnology, Gainesville, FL, USA) 1:1 000 and anti-TH (MAB318, MerckMillipore, Darmstadt, Germany) 1:500. Secondary antibodies included Alexa Fluor 488-conjugated goat anti-rabbit (A-11008, Invitrogen, Thermo Fisher Scientific, Waltham, MA USA) 1:500, Alexa Fluor 555-conjugated goat anti-chicken (ab150170, Abcam, Cambridge, UK) 1:500 and Alexa Fluor 647-conjugated donkey anti-mouse (A-32787, Invitrogen, Thermo Fisher Scientific, Waltham, MA USA) 1:500. Slices were rinsed, incubated in DAPI 1:1 000 (D9542, Merck, Sigma-Aldrich, Darmstadt, Germany) 5 minutes and finally mounted.

***Imaging, cell counting and analysis***

Stained brain sections were imaged using a Nikon A1 laser scanning microscope and the software NIS-Elements AR (version 5.02.01, Nikon Instruments Inc., Tokyo, JP) with 63X and 20X objectives. Confocal images were collected at 512 x 512 pixels with Z steps of 2µm (mPOA) or 0.5µm (VTA) and 488, 647, 564 and 405 lasers. QuPath software (18) was used for cell counting in the regions of interest (ROI). The percentage of Gal^+^ cells was calculated counting the number of neurons positive for galanin (green) over the total number of cells (DAPI) in the MPN and the vBNST of two slices. The percentage of activated Gal^+^ cells was calculated counting the number of cells positive for both Gal and phospho-c-Fos (yellow) over the total number of Gal^+^ cells (green) and compared between groups.

ImageJ-1.53c (Wayne Rasband, National Institutes of Health, USA) software was used for anatomical characterization and neural tracing. mPOA Gal^+^ 🡪 VTA neurons were differentiated in multipolar, bipolar, pyramidal, pseudounipolar, and unknown based on their morphology (19).

Images representing regions of injections (**Supplementary** **Fig. 3 and 5**) were acquired with Zeiss Axiovert 200M Fluorescent/Live cell Imaging Microscope (Carl Zeiss AG, Oberkochen, Germany).

1. **Single unit in vivo electrophysiology recording**

***Microdrive construction***

Microdrives were constructed as previously described (20,21) . Four tetrodes made of twisted 25-um-thick platinum iridium wires (California Wires, USA) were fixed to each exposed wire of a reusable 16 channel microdrive (Axona, UK) and coated with a layer of Pelco conductive silver paint (Ted Pella, Inc., USA). Tetrodes were inserted through a 23-gauge stainless steel cannula and an outer 16-gauge cannula was slipped over the inner cannula and secured via modelling clay. The microdrive was then coated with liquid electrical tape (Gardner Bender, USA). On the surgery day, electrodes were cut to the appropriate length and electroplated with a platinum/gold solution until the impedances dropped within a range of 150–200 KOhms.

***Surgery and tetrodes implantation***

3-5 months old pregnant females were used for single unit in vivo electrophysiology. Females were implanted at ED15, after being either injected with a vehicle solution or immune activated as described above. Isoflurane was administered by a VetFlo Traditional Anaesthesia vaporizer (Kent Scientific) for 1 minute (3-4% Isoflurane concentration): deep anaesthesia was confirmed by the absence of the toe withdrawal reflex. The head of the mouse was fixed in the stereotactic frame (David Kopf Instruments, Tujunga, CA, USA) using ear bars and constant anesthesia was delivered with oxygen flow rate of 1 000 mL per minute and 0.5-3% Isoflurane concentration. As described previously (20,21), an incision was made to expose the skull, and 3 jeweler’s screws were inserted into the skull to support the microdrive implant. A 2-mm hole was made on the skull at coordinates AP=-3.2, ML=0.6. An additional screw connected with wire was inserted into the skull, serving as a ground/reference. The tetrodes were then lowered to the VTA region, 4-4.2 mm from the surface of the brain (below dura). The microdrive ground wire was then soldered to the skull screw wire and the microdrive was secured with dental cement. Pregnant females were then placed on a warm heating pad overnight and monitored constantly.

***In vivo recording and single unit analysis***

Females were habituated to the head stage placement for 10 minutes every day in the experimental room from PD0 to PD3. At PD4, mice were habituated to the experimental room for 1 hour at 19-20 Lux and 22-23 °C. Baseline recording was performed for 10 minutes followed by 15 minutes of pup retrieval assay with 4 foster pups 3-5 days old. Between PD0 and PD3, when required (low number of recorded units), tetrodes were moved down 100µm from their original position (DV=-4, -4.2). Single units were recorded using the Axona DacqUSB system as described previously (20,21). Recording signals were amplified 10 000 to 30 000 times and bandpass filtered between 0.3 and 7KHz (22). In order to record longer spikes, Tint mode was set to 2ms and sampling rate to 24kHz as recommended in the manual of the Axona DacqUSB system. A Notch filter was applied to remove electrical noise (60Hz). Offline spike sorting was done using Tint cluster-cutting software and KlustaKwik automated clustering, followed by further manual cleaning: clusters with less than 200 spikes and same amplitude in each channel were discarded from the analysis. Quantitative measurements of cluster quality were then performed, yielding isolation distance values in Mahalanobis space (23). Median isolation distance values were then calculated per mouse and compared across treatments. There were no significant differences between clusters (isolation distances: vehicle, 6.68; Poly I:C, 4.94; t_(8)_ = 1.555; P = 0.159; N = 5 animals/group).

Used automated analysis tools were SpikeAnalysis GUI, SpikeWidth GUI, Spiketimesplot, Raster GUI (<https://github.com/HussainiLab> available upon request) and FRATE from Axona.

-SpikeWidthGUI: we used this python-based Graphic User Interface (GUI) to analyse the spike width of the neuronal waveforms. It also calculates other spike properties such as the Average (filtered/unfiltered) Firing Rates and Peak (filtered/unfiltered) Firing Rates.

-SpikeAnalysisGUI: we used this python-based GUI to quantify spike sorting quality by generating the Isolation distance between the neuronal clusters. It also analysed bursting activity and ISI of the neurons.

-The MATLAB-script, Spiketimesplot and Tint-tool, FRATE were used to calculate the firing rate during specific times.

-Raster GUI python-script was used to calculate spike times between specific time points.

For baseline analysis between vehicle and Poly I:C injected mice, individual neuronal firing rates (FR) were calculated by dividing the total number of spikes per neuron by the duration of the baseline session (10’). For the analysis during pup retrieval, the FR was calculated during three behavioral epochs, pup approach, pup retrieval and post interaction interval and normalized to average baseline FR: since no difference in FR was detected between first and last 5 minutes of baseline recording and during stillness or movement, average baseline FR was calculated along the entire session.

The activity change for each neuron was calculated using the standardized change ratio (delta FR):

(FR behavior – FR baseline)/ (FR behavior + FR baseline)

where behavior corresponds to one of the three behavioral epochs.

Pup approach was identified as the time between the first step outside the nest towards the pups and the start of the pup retrieval; pup retrieval was identified as the time between the mother makes clear contact with the pup and the pup is released into the nest (17). Pup approach and pup retrieval FRs were calculated as the average between the FRs during the first and the last pup approach and first and last pup retrieved: times ranged between 2ms and 6ms. Post interaction was identified as the average between three time points after the last pup is retrieved in the nest: 10 sec immediately after, 10 sec one minute after and 10 sec two minutes after the last pup is retrieved. Mice that did not retrieve the pups were excluded by the pup retrieval analysis.

***Classification of neurons***

A total of 136 VTA neurons were recorded from 10 mice (vehicle, N = 60 neurons; Poly I:C, N = 76 neurons). Neurons were first divided into fast firing neurons and slow firing neurons based on their average FR measured during baseline. FR were calculated averaging the individual FR per group (vehicle or Poly I:C). Neurons with FR > 10Hz were classified as fast firing neurons while neurons with FR < 10Hz were classified as slow firing neurons. Spike width was calculated as full action potential duration, from the initial inflection to the return to baseline. Based on the frequency distribution histogram of pooled single-unit spike widths, narrow spiking neurons were considered as neurons having spike width < 1200µs and wide spiking neurons as neurons having spike width > 1400µs. Neurons having total action potential width between 1200µs and 1400µs were assigned to either group based on clear waveform characteristics or excluded from the analysis. Most wide spiking neurons had clear putative dopaminergic characteristics: long action potential duration, FR < 10Hz, presence of a large negative undershoot due to calcium component and slow depolarization (22,24–28) (vehicle, N = 16 neurons; average FR during baseline= 2.55 ± 1.84; average spike width= 1889 ± 38.6 µs). Moreover, most of putative dopaminergic neurons showed burst episodes: a burst was defined as having 2 spikes with ISI < 80ms and total burst duration not exceeding 160ms (29) (vehicle, average bursting percentage= 46% ± 5.8; average spikes/burst= 2.5 ± 0.1). Narrow spiking neurons showed narrow peak width, short action potential duration and FR < 10Hz. We considered this population as putative GABAergic neurons (vehicle, N = 21 neurons; average FR during baseline=1.54 ± 0.24; average spike width= 874 ± 60.3 µs) (22,24,25,30). Fast firing neurons had FR > 10Hz, narrow peak width and short action potential duration (vehicle, N = 8 neurons; average FR during baseline= 15.75 ± 2.23; average spike width= 798 ± 70.8 µs). Fast-firing neurons were only detected in some of the animals (5 out of 10 animals) and therefore were excluded from further analysis.

1. **Statistics**

All analyses were performed by an investigator blinded to the experimental treatment of mice. N numbers, full statistics and Ρ values are reported for each main effect and all interactions are listed where relevant. Sample sizes were selected based upon own experience and on data available in literature (11,31,32). Statistical outliers were calculated with the online available Graphpad outlier calculator, using α = 0.05 as significance level. Normality was assessed with Shapiro-Wilk test: where not normal, data were analysed with non-parametric tests. Levene’s test was used to test equality of variances (with Welch correction, if equal variances were not assumed). Mann-Whitney U test, Student’s t- test and Spearman correlation were used for all behavioral analysis and for c-Fos staining analysis; statistical analysis of the neuronal morphology and spine density was done with a two-way ANOVA and Mixed ANOVA was used for Sholl analysis. For Sholl analysis, data was log transformed. Since assumption of sphericity was violated, degrees of freedom were corrected using the Greenhouse Geisser estimates of sphericity. In vivo electrophysiology data were analysed with Student’s t test, mixed ANOVA and Mann-Whitney test if not normal. Additionally, a pairwise comparison was performed as Post Hoc analysis in cases of an interaction between two groups.

Graphs were made with GraphPad Prism 7.0 software (San Diego, CA, USA).

**References**

1. Khan D, Fernando P, Cicvaric A, Berger A, Pollak A, Monje FJ, et al. Long-term effects of maternal immune activation on depression-like behavior in the mouse. Transl Psychiatry. 2014 Feb 18;4:e363.

2. Van der Lee S, Boot LM. Spontaneous pseudopregnancy in mice. Acta Physiol Pharmacol Neerl. 1955;4(3):442–4.

3. Whitten WK. Modification of the oestrous cycle of the mouse by external stimuli associated with the male. J Endocrinol. 1956;13(4):399–404.

4. Franks B, Curley JP, Champagne FA. Measuring Variations in Maternal Behavior: Relevance for Studies of Mood and Anxiety. Springer. 2011;63:209–24.

5. Ronovsky M, Berger S, Zambon A, Reisinger SN, Horwath O, Pollak A, et al. Maternal immune activation transgenerationally modulates maternal care and offspring depression-like behavior. Brain Behav Immun. 2017 Jul;63:127–36.

6. Kreitz S, Zambon A, Ronovsky M, Budinsky L, Helbich TH, Sideromenos S, et al. Maternal immune activation during pregnancy impacts on brain structure and function in the adult offspring. Brain Behav Immun. 2020;83:56–67.

7. Berger S, Ronovsky M, Horvath O, Berger A, Pollak DD. Impact of maternal immune activation on maternal care behavior, offspring emotionality and intergenerational transmission in C3H/He mice. Brain, behavior, and immunity. 2018;70:131–40.

8. Kentner A, Bilbo S, Brown A, Hsiao EY, Mcallister A, Meyer U, et al. Maternal immune activation: reporting guidelines to improve the rigor, reproducibility, and transparency of the model. 2019 Jan;245–58.

9. Gandhi R, Hayley S, Gibb J, Merali Z, Anisman H. Influence of poly I:C on sickness behaviors, plasma cytokines, corticosterone and central monoamine activity: Moderation by social stressors. Brain Behav Immun. 2007;21(4):477–89.

10. Arsenault D, St-Amour I, Cisbani G, Rousseau L-S, Cicchetti F. The different effects of LPS and poly I:C prenatal immune challenges on the behavior, development and inflammatory responses in pregnant mice and their offspring. Brain Behav Immun. 2014;38:77–90.

11. Kohl J, Babayan BM, Rubinstein ND, Autry AE, Marin-Rodriguez B, Kapoor V, et al. Functional circuit architecture underlying parental behaviour. Nature. 2018;556(7701):326–31.

12. Scattoni ML, Gandhy SU, Ricceri L, Crawley JN. Unusual repertoire of vocalizations in the BTBR T+tf/J mouse model of autism. PLoS One. 2008;3(8):e3067.

13. Paxinos G, Franklin KBJ. The mouse brain in stereotaxic coordinates. In: Second. Academic Press; 2001.

14. Gerecke KM, Kishore R, Jasnow A, Quadros-Menella P, Parker S, Kozub FJ, et al. Alterations of Sex-Typical Microanatomy: Prenatal Stress Modifies the Structure of Medial Preoptic Area Neurons in Rats. Dev Psychobiol. 2012;54(1):16–27.

15. Bogus-Nowakowska K, Robak A, Szteyn S, Równiak M, Najdzion J, Wasilewska B. The neuronal structure of the preoptic area in the mole and the rabbit: Golgi and Nissl studies. Folia Morphol. 2006;65(4):367–76.

16. Risher WC, Ustunkaya T, Alvarado JS, Eroglu C. Rapid Golgi analysis method for efficient and unbiased classification of dendritic spines. PLoS One. 2014;9(9):e107591.

17. Fang YY, Yamaguchi T, Song SC, Tritsch NX, Lin D. A Hypothalamic Midbrain Pathway Essential for Driving Maternal Behaviors. Neuron. 2018;98(1):192–207.

18. Bankhead P, Loughrey MB, Fernández JA, Dombrowski Y, McArt DG, Dunne PD, et al. QuPath: Open source software for digital pathology image analysis. Sci Rep. 2017;7(1):16878.

19. Kandel ER, Schwartz JH, Jessell TM. Principles of Neural Science. 4th ed. McGraw-Hill, NY, US: McGraw-Hill Companies, Inc.; 2000.

20. Fu H, Rodriguez GA, Herman M, Emrani S, Nahmani E, Barrett G, et al. Tau Pathology Induces Excitatory Neuron Loss, Grid Cell Dysfunction, and Spatial Memory Deficits Reminiscent of Early Alzheimer’s Disease. Neuron. 2017;93(3):533–41.

21. Rodriguez GA, Barrett GM, Duff KE, Hussaini SA. Chemogenetic attenuation of neuronal activity in the entorhinal cortex reduces Aβ and tau pathology in the hippocampus. PLoS Biol. 2020;18(8):e3000851.

22. Li W, Doyon WM, Dani JA. Quantitative unit classification of ventral tegmental area neurons in vivo. J Neurophysiol. 2012;107(10):2808–20.

23. Schmitzer-Torbert N, Jackson J, Henze D, Harris K, Redish AD. Quantitative measures of cluster quality for use in extracellular recordings. Neuroscience. 2005;131(1):1–11.

24. Ungless MA, Grace AA. Are you or aren’t you? Challenges associated with physiologically identifying dopamine neurons. Trends Neurosci. 2012;35(7):422–30.

25. Mohebi A, Pettibone JR, Hamid AA, Wong J-MT, Vinson l. T, Patriarchi T, et al. Dissociable dopamine dynamics for learning and motivation. Nature. 2019;570(7759):65–70.

26. Brischoux F, Chakraborty S, Brierley DI, Ungless MA. Phasic excitation of dopamine neurons in ventral VTA by noxious stimuli. Proc Natl Acad Sci U S A . 2009;106(12):4894–9.

27. Burkhardt JM, Adermark L. Locus of onset and subpopulation specificity of in vivo ethanol effect in the reciprocal ventral tegmental area–nucleus accumbens circuit. Neurochem Int . 2014;76:122–30.

28. Grace AA, Bunney BS. Intracellular and extracellular electrophysiology of nigral dopaminergic neurons. Identification and characterization. Neuroscience. 1983;10(2):301–15.

29. Grace AA, Bunney BS. The control of firing pattern in nigral dopamine neurons: burst firing. J Neurosci. 1984;4(11):2877–90.

30. Tan KR, Yvon C, Turiault M, Mirzabekov JJ, Doehner J, Labouèbe G, et al. GABA neurons of the VTA drive conditioned place aversion. Neuron. 2012;73:1173–83.

31. Keyser-Marcus L, Stafisso-Sandoz G, Gerecke K, Jasnow A, Nightingale L, Lambert KG, et al. Alterations of medial preoptic area neurons following pregnancy and pregnancy-like steroidal treatment in the rat. Brain Res Bull. 2001;55(6):737–45.

32. Wu Z, Autry AE, Bergan JF, Watabe-Uchida M, Dulac CG. Galanin neurons in the medial preoptic area govern parental behaviour. Nature. 2014;509(7500):325–30.
