# Supplementary material for: Gestational immune activation disrupts hypothalamic neurocircuits of maternal care behavior
Source: Mol Psychiatry. 2022 May 17;29(4):859–73. doi: 10.1038/s41380-022-01602-x (PMC9112243; doi:10.1038/s41380-022-01602-x)
Supplement: Supplementary file 2 — Supplementary Tables [file 41380_2022_1602_MOESM2_ESM.docx]

# **Supplementary Tables**

## **Supplementary Table 1.** **Classification of different call types of pup USVs.**

Pup ultrasonic vocalizations (USVs) were manually marked from the recorded sonogram using UltraVox XT software (Noldus, Wageningen, the Netherlands) and classified based upon duration and shape features. ms = milliseconds

| **Call name** | **Shape** | **Duration** |
| --- | --- | --- |
| Short | 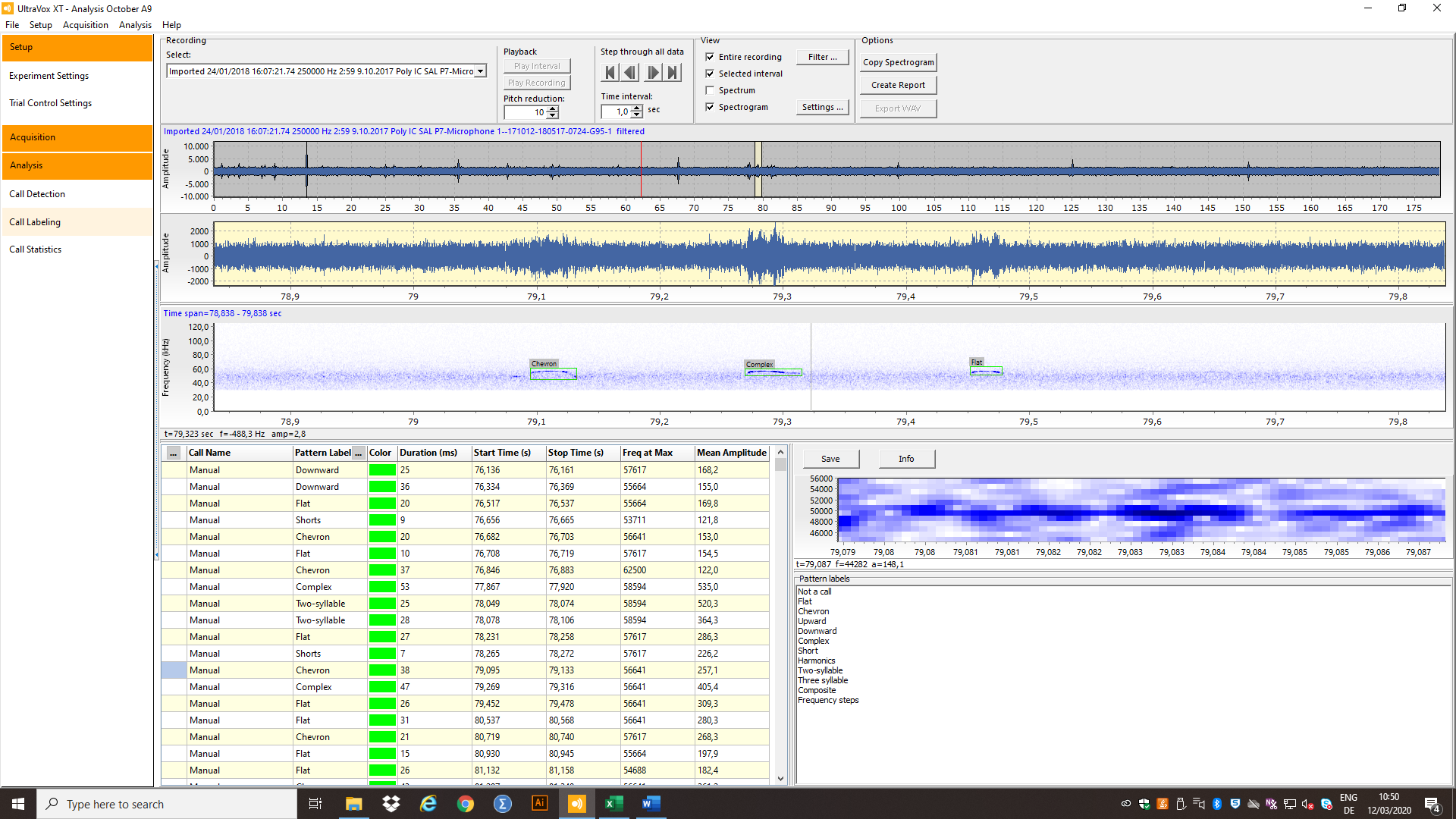 | </= 10 ms |
| Flat | 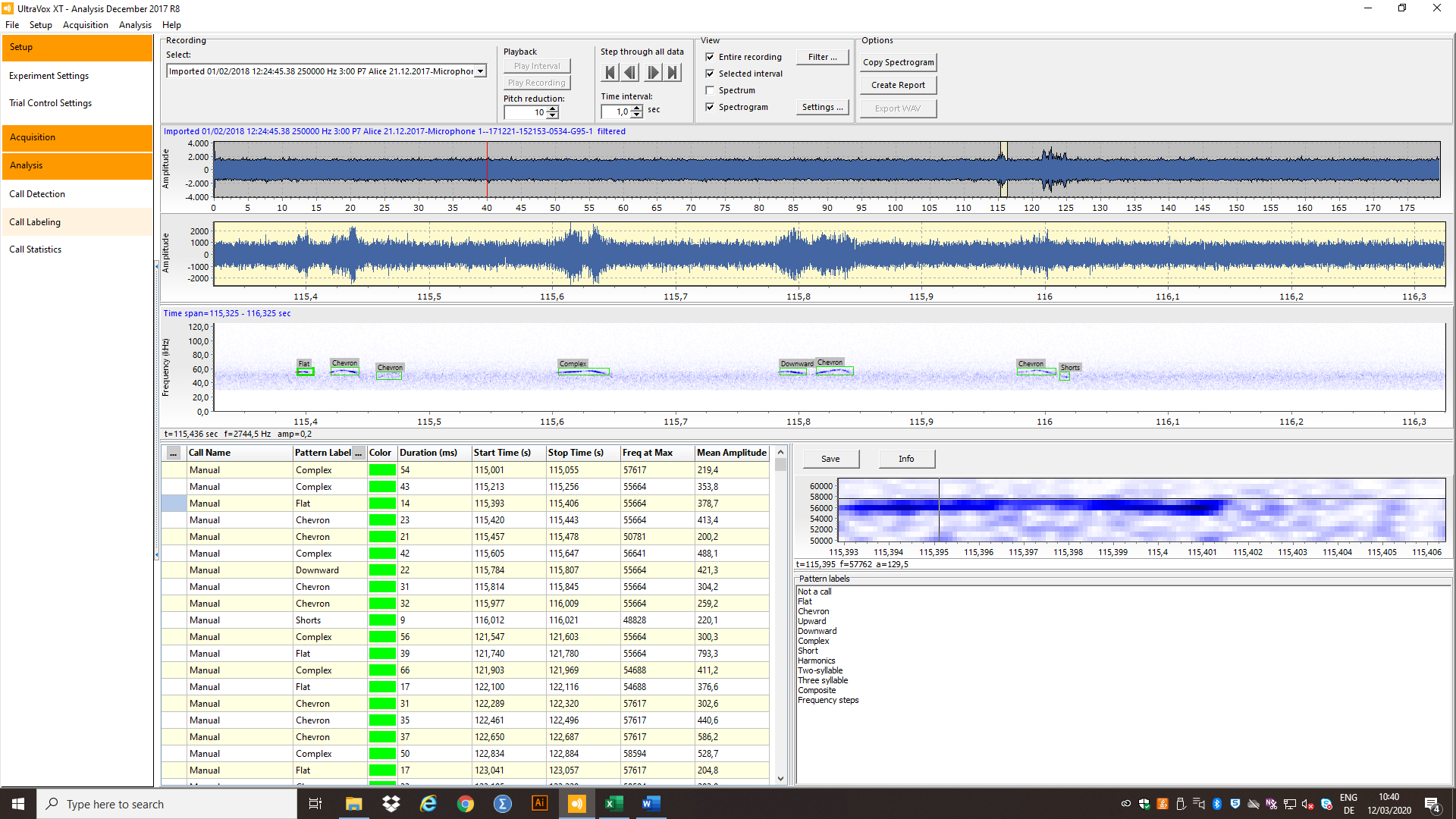 | 10 ms < x < 40 ms |
| Upward | 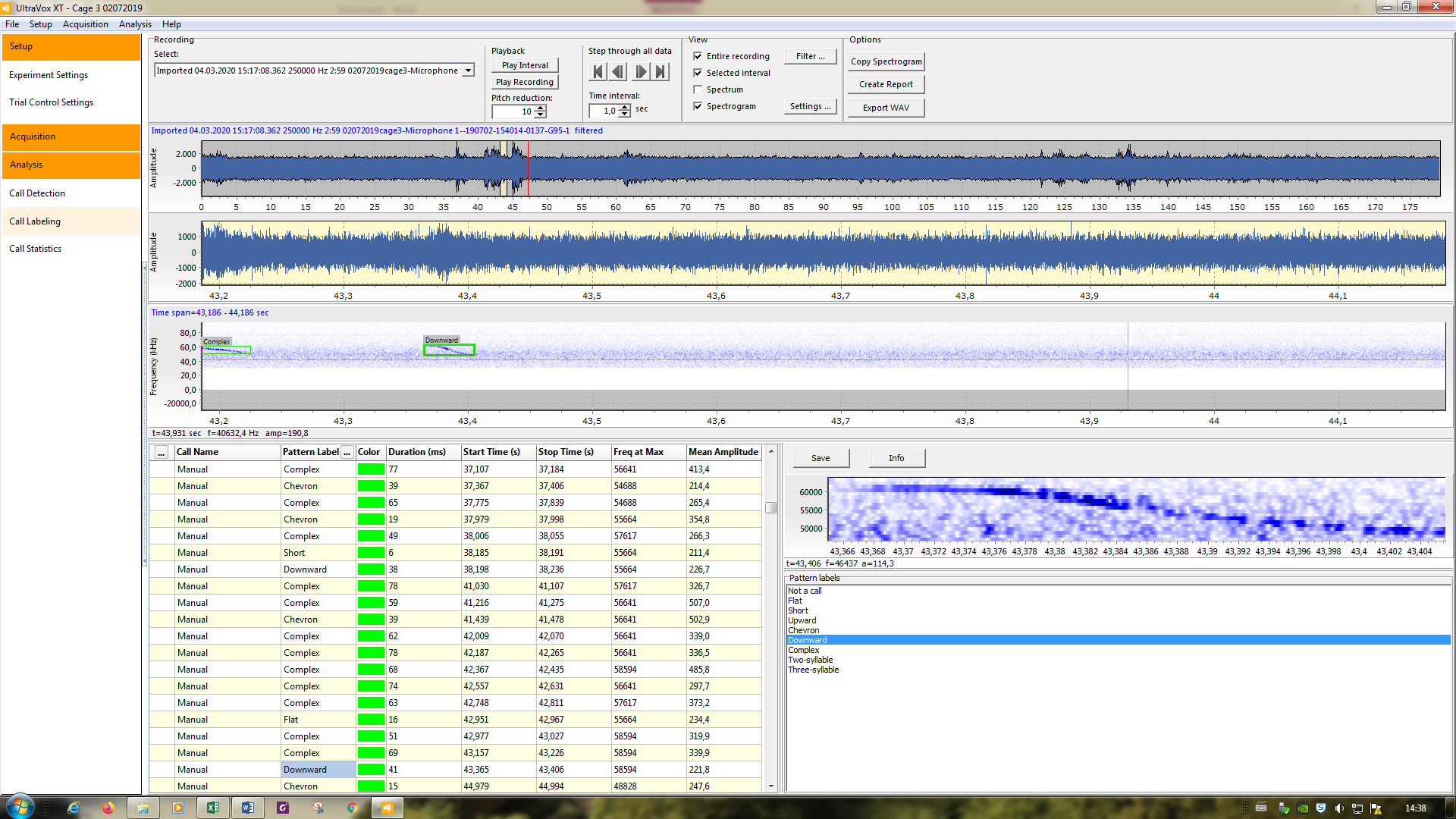 | 9 ms < x < 40 ms |
| Downward | 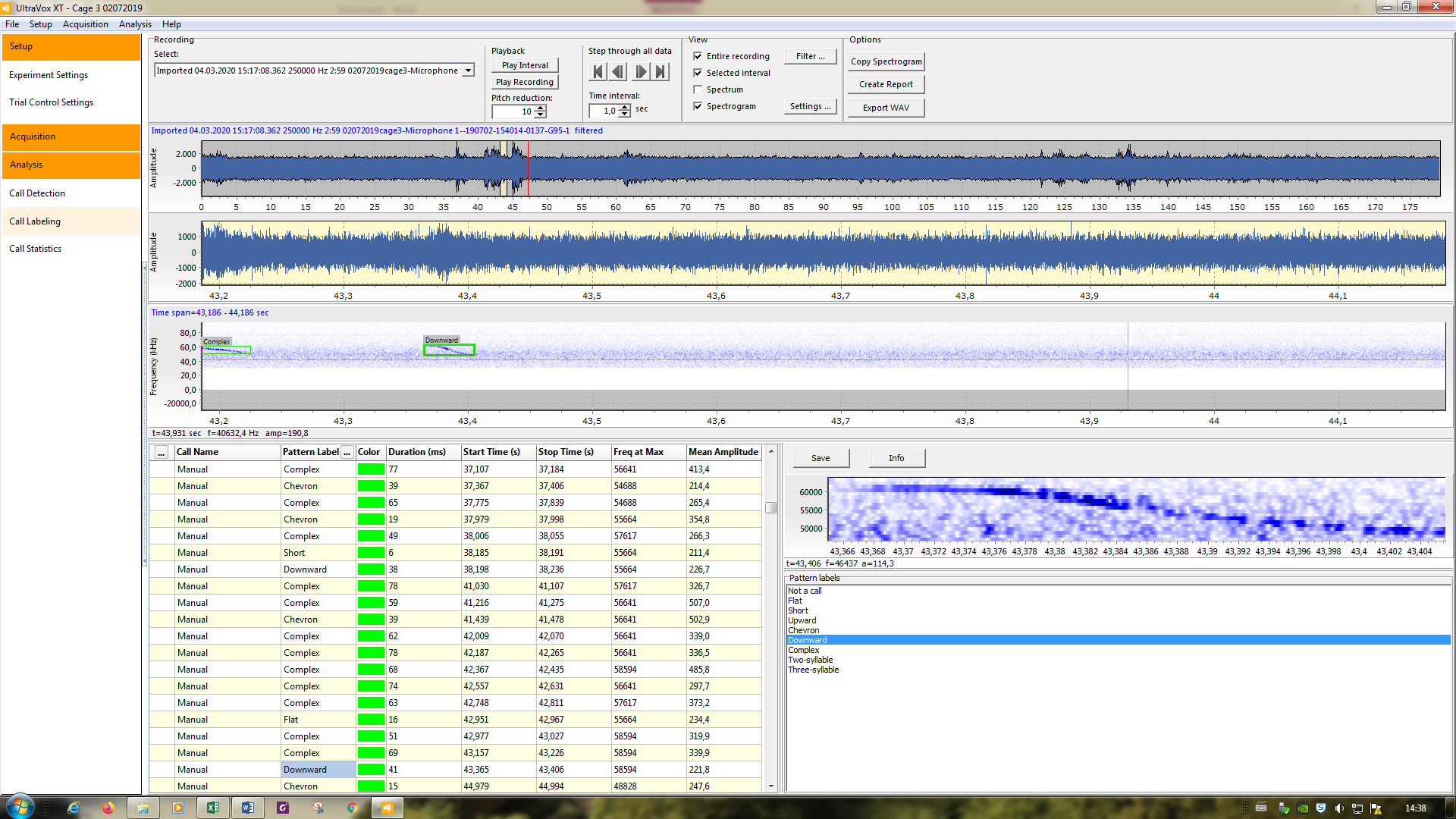 | 10 ms < x < 50 ms |
| Chevron | 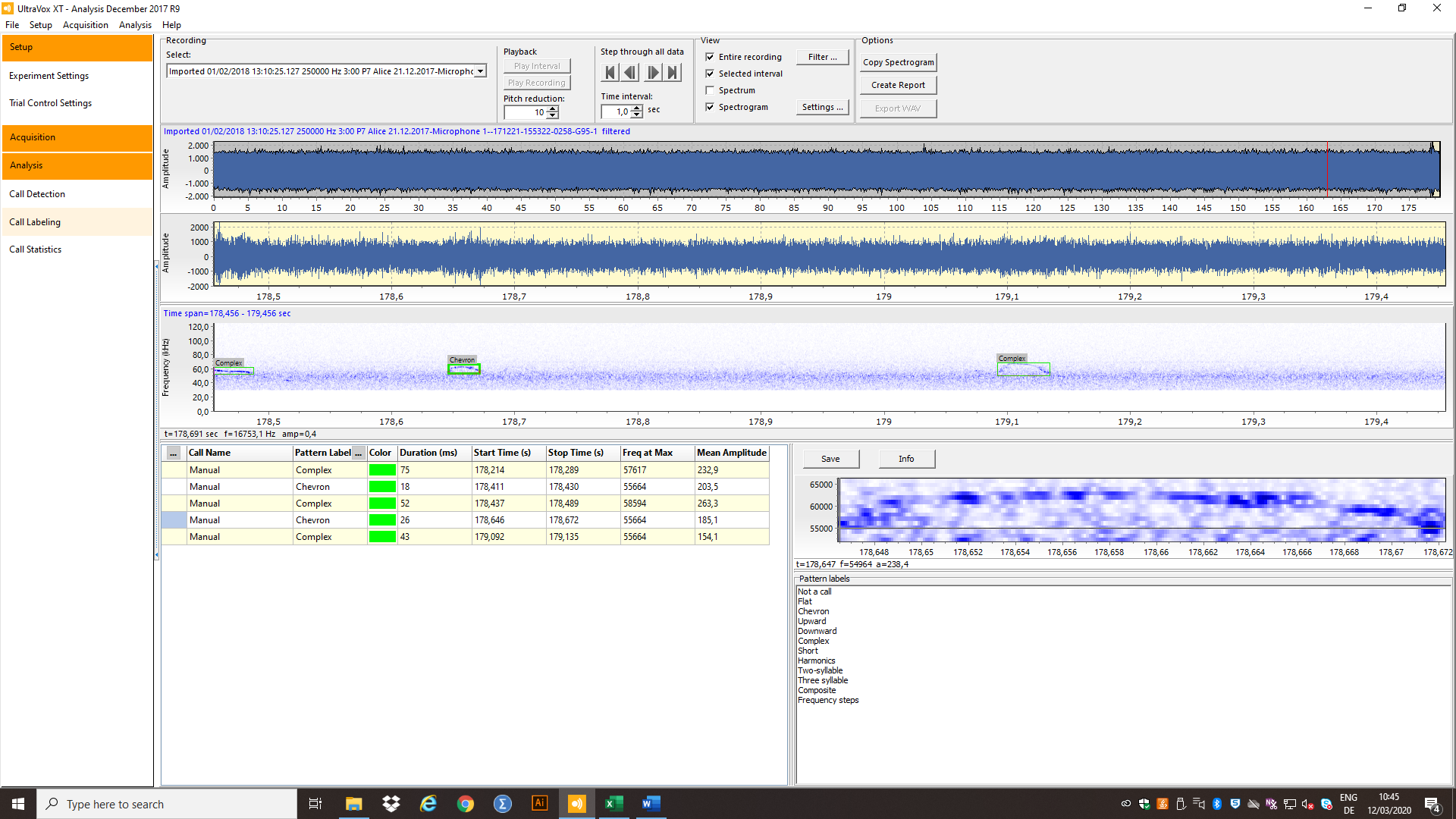 | 10 ms < x < 40 ms |
| Complex | 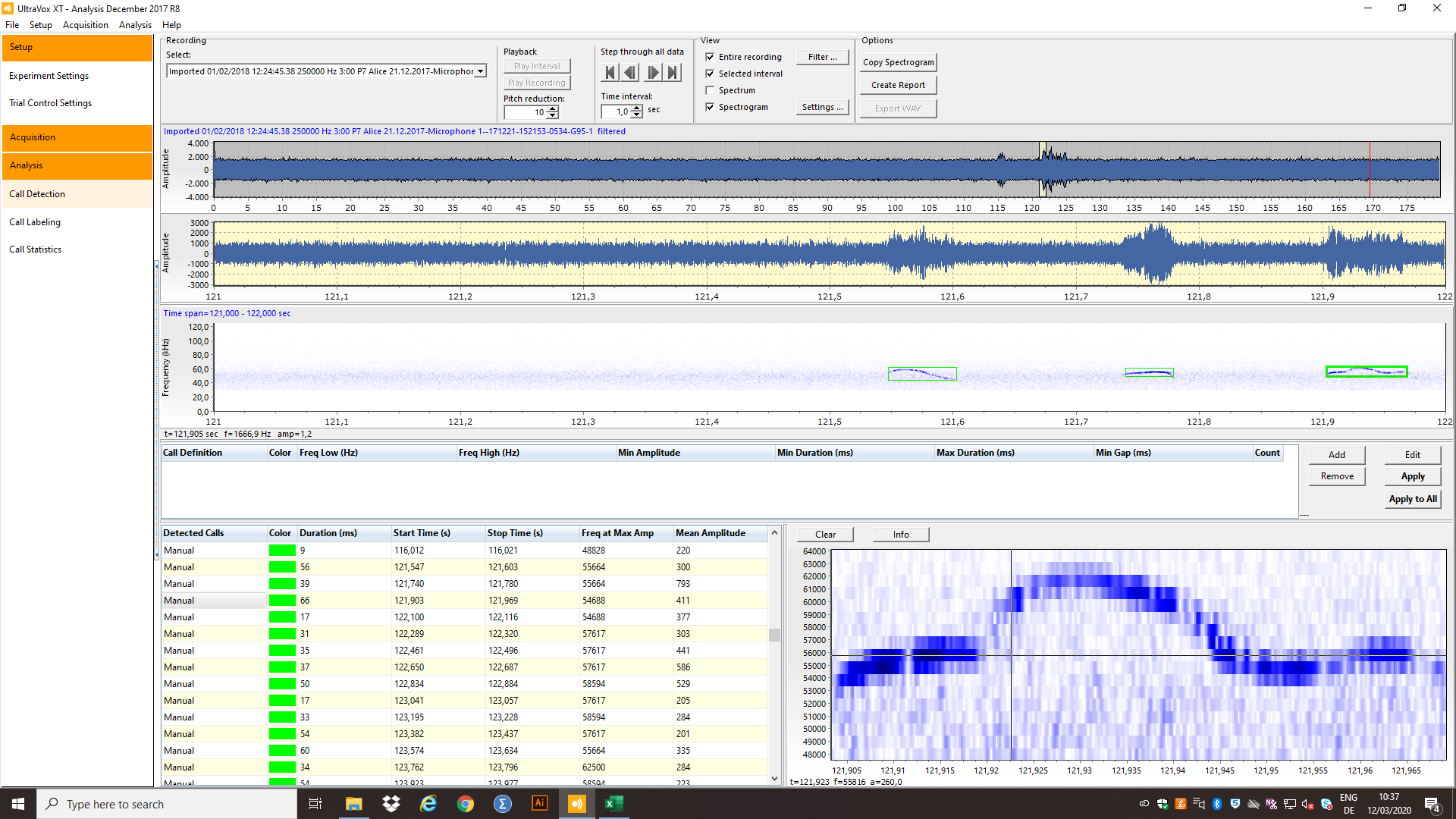 | >/= 40ms |

## **Supplementary Table 2. Transgenic lines and viruses used.**

| **Name given in the test** | **Stock number** | **Name of transgenic line** | **Function** |
| --- | --- | --- | --- |
| *Gal::Cre* | Stock no 036969-UCD  MMRRC North America | B6.FVB(Cg)-Tg(Gal-cre)KI87Gsat/Mmucd, | Cre recombinase is expressed under the control of the galanin promoter |
| *FlexEYFP* | JAX stock 006148  (Jackson Laboratories, Bar Harbour, ME, USA) | B6.129X1-Gt(ROSA)26Sortm1(EYFP)Cos/J | EYFP expression is directed to cells expressing the Cre recombinase |
| *vGlut::Flp* | JAX stock 030212  (Jackson Laboratories, Bar Harbour, ME, USA) | B6;129S-Slc17a6tm1.1(flpo)Hze/J | Flp recombinase expression is directed to vGLUT2 expressing cells |
| *vGat::Flp* | JAX stock 029591  (Jackson Laboratories, Bar Harbour, ME, USA) | B6.Cg-Slc32a1tm1.1(flpo)Hze/J | Flp recombinase expression is directed to vGAT expressing cells |

| **Name given in the text** | **Catalogue number** | **Name of the virus** | **Function** |
| --- | --- | --- | --- |
| AAV-FLEx-syn1-EGFP | AAV9 100043  Addgene, Massachusetts, USA | pAAV.synP.DIO.EGFP.WPRE.hGH | Cre dependent AAV  directing the expression of EGFP |
| AAV-FRT-Ef1a-tdTomato | Vector Core, IST, Klosterneuburg, Austria |  | Flp dependent AAV directing the expression of tdTomato |
| AAVrg-FLEx-CAG-tdTomato | Addgene 28306-AAVrg  Addgene, Massachusetts, USA | pAAV-FLEX-tdTomato | Cre dependent retrograde AAV directing the expression of tdTomato |
| AAVrg-FLEx-hsyn-EGFP | Addgene 50457 AAVrg  Addgene, Massachusetts, USA | pAAV-hSyn-DIO-EGFP | Cre dependent retrograde AAV directing the expression of EGFP |
| AAVrg-FRT-Ef1a-mCherry | Addgene 114471 AAVrg  Addgene, Massachusetts, USA | pAAV-Ef1a-fDIO mCherry | Flp dependent retrograde AAV directing the expression of mCherry |

## **Supplementary Table 3. MIA model reporting guidelines checklist.**

A MIA model checklist was created according to the guidelines provided by Kentner and colleagues (1).

| **Study design** ➢ Overview of immune activation issues | |
| --- | --- |
| Compounds | -Name of compound: Polyinosinic:polycytidylic acid (Sigma-Aldrich)  -Catalogue number: P9582  -Lots number: #096M4023V, #117M4005V, #118M4035V, #059M4167V  -Vehicle control used: 0.9% physiological NaCl  -Route of administration: intraperitoneal  -Volume administered: 20mg/kg (10uL/g)  -Storage conditions: -20°C  -Anaesthetics (type, dose, duration) used: isoflurane (2‐2.5%) in air (2 l/min); ketamine 25mg/mL; xylazine 10mg/mL (lethal dosage) |
| Housing variables at injection | -Temperature of room at injection time: 22,5 °C  -Light cycle of animal housing room: light/dark 12:12  -Time of day of injection: 8:30-9:30 h  -No cage change at time of injection |
| Validation of immune activation | IL-6 ELISA and sickness behavior scoring respectively 3h and 2h after injection |
| Validation of gestational timing | Timed mating and vaginal plug |
| **Experimental animals** | |
| Species | Mouse |
| Strain | C57Bl/6N and C57Bl/6J (*transgenic* lines) |
| Maternal/Offspring Physiological Variables at time of immune challenge – age, body weight | -Maternal Age at challenge: 11/16 weeks  -Maternal Body weight at challenge: ~29-30g on average |
| Vendor | Charles River Laboratories and Jackson Laboratory  -Location of Vendors: Austria / Germany / United States (New York)  -Room/area where animals originated from: N/A |
| Caging systems | -During mating: Maxi miser caging system  plastic cage dimensions: 19.56 x 30.91 x 13.34 cm  -During pregnancy: open cages  plastic cage dimensions: 19.56 x 30.91 x 13.34 cm |
| Animal Holding Room | -Temperature: 21-23 °C  -Humidity: ~ 40%  -Ventilation system: IVC or open cages  -Conventional animal room  -Males and females are housed in the same room, but separate areas |
| Bedding exchanges/ bedding type | -Premium scientific bedding (SAFE)  -Cage changes: once a week  during gestation: twice  during neonatal period: no cleaning till experiment (PD4 or PD7) |
| Breeding | -Timed pregnancy  -Same fathers are bred with both experimental and control dams when both dams are pregnant  -Breeding location: IVC Maxi miser caging system and open cages  -Biological age at shipping: 7/8 weeks  -Biological age of dams: 9/12 weeks (all experiments), 10/20 weeks (in vivo electrophysiology)  -Number of times dams have been mated previously: 0-2 times  -Number of times the dams mated and not became pregnant: 0-2 times  -Dams are primiparous  -Frequency of maternal handling during the gestational/neonatal period: twice a week  -Biological age of sires: 9/24 weeks  -Number of times sires have been mated previously: 0-6 times  -Number of times the sires mated successfully: between 1 and 3 times  -If bred previously, interval between mating times: one week  -Are sires matched to experimental and control dams? yes  -Mating design: 1:2 |
| Social enrichment | -Number of cage companions prior to breeding: 3-5  -Gestational age when dam separated for breeding: 9/14 weeks |
| Physical enrichment | -Enrichment devices included in the cage: 1 paper towel |
| **Sample size** ➢ Litter versus offspring | |
| Maternal- N vs offspring N | -Total number of -dams/litters included in the study: 97  -Total number of offspring per litter included in the study: 84 pups (USVs) |
| Litter size and sex distribution | -Litter size: 6-10 for WT mice, 4-10 for transgenic mice |
| Cross fostering | -Foster pups were used for in vivo electrophysiology experiments |
| **Allocating animals to experimental groups** | |
| How many offspring per litter were used in each measure |  |
| Randomization/Matching procedures | Procedure used to assign animals to groups: randomization |
| Sex as a biological variable (behavioral and physiological outcomes) | Mothers and pups from both sexes (USVs) |
| **Experimental outcomes** ➢ Behavioral testing ➢ Physiological endpoints | |
| Order of testing (e.g. behavioral test order) | All animals were evaluated in a counter-balanced order in terms of order of experimental/control groups |
| **Statistical methods** |  |
| Unit of analysis for each data set | The unit (Ν) of each analysis was based on number of dams or number of neurons (in vivo electrophysiology) |

## **Supplementary Table 4. Sickness behavior scoring.**

The severity of sickness behavior in Poly I:C versus vehicle injected mice was evaluated assigning a score between 0 and 3 by for the following parameters: body position, ptosis, piloerection, and nest condition. Presence/ absence of diarrhea was also recorded (2,3).

| **Sickness behavior scoring** | |
| --- | --- |
| Body posture | 0 normal sitting up or rearing on hind paws  1 slightly curled body posture  2 curled body posture  3 lethargy curled body posture |
| Ptosis (dropping eye lids) | 0 eyes fully open  1 slightly closed  2 1/4 closed  3 1/2 closed or fully closed |
| Piloerection | 0 none / fur flat and tidy  1 slightly at the nape  2 clear piloerection on some parts of the neck  3 extreme piloerections |
| Nest condition (cage) | 0 intact / well defined nest area  1 intact / not well shaped  2 not intact / slightly spread  3 completely spread and disrupted |
| Diarrhea | Yes  not |

## **Supplementary Table 5. Statistical analysis of different call types of pup USVs.**

USVs were classified as *flat, short, upward, downward, chevron and complex*. Mean amplitude, mean duration, mean frequency, and number of calls per litter were analysed for each call category between Poly I:C and control groups. N refers to number of mothers per group. Student’s t tests were used for statistical analysis.

| **Call type** | **Parameter** | **Statistics** |
| --- | --- | --- |
| Short | Mean amplitude | t (18) = -1.684, Ρ = 0.109, Ν = 10,10 |
| Short | Mean duration | t (18) = -0.271, Ρ = 0.790, Ν = 11,9 |
| Short | Mean frequency | t (18) = 1.025, Ρ = 0.319, Ν = 11,9 |
| Short | Number of calls/litters | t (19) = 0.403, Ρ = 0.691, Ν = 11,10 |
| Flat | Mean amplitude | t (18) = -1.810, Ρ = 0.087, Ν = 10,10 |
| Flat | Mean duration | t (18) = 2.308, Ρ = **0.033**, Ν = 11,9 |
| Flat | Mean frequency | t (19) = -0.110, Ρ = 0.913, Ν = 11,10 |
| Flat | Number of calls/litters | t (19) = 0.835, Ρ = 0.414, Ν = 11,10 |
| Upward | Mean amplitude | t (18) = -0.103, Ρ = 0.919, Ν = 11,9 |
| Upward | Mean duration | t (17) = 0.748, Ρ = 0.465, Ν = 10,9 |
| Upward | Mean frequency | t (16) = 1.117, Ρ = 0.281, Ν = 10,8 |
| Upward | Number of calls/litters | t (9.867) = -1.051, Ρ = 0.318, Ν = 11,9 |
| Downward | Mean amplitude | t (18) = -0.774, Ρ = 0.449, Ν = 10,10 |
| Downward | Mean duration | t (18) = 0.256, Ρ = 0.801, Ν = 10,10 |
| Downward | Mean frequency | t (19) = 1.237, Ρ = 0.231, Ν = 11,10 |
| Downward | Number of calls/litters | t (19) = 0.467, Ρ = 0.646, Ν = 11,10 |
| Chevron | Mean amplitude | t (12.805) = -0.146, Ρ = 0.886, Ν = 11,10 |
| Chevron | Mean duration | t (18) = 0.479, Ρ = 0.638, Ν = 10,10 |
| Chevron | Mean frequency | t (19) = 1.134, Ρ = 0.271, Ν = 11,10 |
| Chevron | Number of calls/litters | t (17) = -0.990, Ρ = 0.336, Ν = 10,9 |
| Complex | Mean amplitude | t (18) = -1.284, Ρ = 0.215, Ν = 10,10 |
| Complex | Mean duration | t (19) = 1.765, Ρ = 0.094, Ν = 11,10 |
| Complex | Mean frequency | t (17) = 0.770, Ρ = 0.452, Ν = 10,9 |
| Complex | Number of calls/litters | t (18) = 0.336, Ρ = 0.741, Ν = 11,9 |

## **Supplementary Table 6. Pilot analysis of neuronal morphology of pyramidal and bipolar neurons in the mPOA.**

Neurons in the mPOA were classified in three main subpopulations including bipolar, pyramidal, and multipolar neurons.

| **Neuronal subpopulation** | **Parameter analysed** | **Statistics** |
| --- | --- | --- |
| Bipolar neurons | Cumulative length | t (12) = 1.169, Ρ = 0.265, Ν = 6, 8 neurons/ group |
|  | Number of nodes | t (13) = 0.668, Ρ = 0.516, Ν = 6, 9 neurons/ group |
|  | Cell body area | t (12) = 0.287, Ρ = 0.779, Ν = 6, 8 neurons/ group |
| Pyramidal neurons | Cumulative length | t (12) =-0.759, Ρ = 0.463, Ν = 8, 6 neurons/ group |
|  | Number of nodes | t (12) =-1.329, Ρ = 0.208, Ν = 8, 6 neurons/ group |
|  | Cell body area | t (13) =-0.680, Ρ = 0.509, Ν = 9, 6 neurons/ group |

## **Supplementary Table 7. Analysis of dendritic spines density and neuronal morphology in the VTA.**

Spines were categorized into *filopodia, mushroom, stubby, thin, and long thin* based on Risher and colleagues algorithm (4). N refers to number of females per group.

| **Parameter analysed** | **Statistical test** | **Statistics** |
| --- | --- | --- |
| Density of spines/10µm | Two-way ANOVA | Main effect of treatment: F (1) = 0.231; Ρ = 0.637; Ν = 5/ group  Main effect of lactation: F (1) = 1.377; Ρ = 0.258; Ν = 5/ group  Interaction treatment*lactation: F (1) = 0.074; Ρ = 0.789; Ν = 5/ group |
| Density of filopodia/10µm | Two-way ANOVA | Main effect of treatment: F (1) = 0.002; Ρ = 0.962; Ν = 5/ group  Main effect of lactation: F (1) = 1.077; Ρ = 0.315; Ν = 5/ group  Interaction treatment*lactation: F (1) = 0.972; Ρ = 0.339; Ν = 5/ group |
| Density of mushroom spines/10µm | Two-way ANOVA | Main effect of treatment: F (1) = 0.599; Ρ = 0.599; Ν = 5/ group  Main effect of lactation: F (1) = 0.095; Ρ = 0.762; Ν = 5/ group  Interaction treatment*lactation: F (1) = 3.628; Ρ = 0.075; Ν = 5/ group |
| Density of stubby spines/10µm | Two-way ANOVA | Main effect of treatment: F (1) = 2.642; Ρ = 0.124; Ν = 5/ group  Main effect of lactation: F (1) = 1.406; Ρ = 0.253; Ν = 5/ group  Interaction treatment*lactation: F (1) = 0.107; Ρ = 0.748; Ν = 5/ group |
| Density of thin spines/10µm | Two-way ANOVA | Main effect of treatment: F (1) = 0.271; Ρ = 0.610; Ν = 5/ group  Main effect of lactation: F (1) = 0.067; Ρ = 0.799; Ν = 5/ group  Interaction treatment*lactation: F (1) = 1.913; Ρ = 0.186; Ν = 5/ group |
| Density of long thin spines/10µm | Two-way ANOVA | Main effect of treatment: F (1) = 0.698; Ρ = 0.416; Ν = 5/ group  Main effect of lactation: F (1) = 1.751; Ρ = 0.204; Ν = 5/ group  Interaction treatment*lactation: F (1) = 0.262; Ρ = 0.616; Ν = 5/ group |
| Neuronal cumulative length | Two-way ANOVA | Main effect of treatment: F (1) = 1.185; Ρ = 0.293; Ν = 5/ group  Main effect of lactation: F (1) = 1.266; Ρ = 0.277; Ν = 5/ group  Interaction treatment*lactation: F (1) = 0.259; Ρ = 0.618; Ν = 5/ group |
| Number of nodes | Two-way ANOVA | Main effect of treatment: F (1) = 0.206; Ρ = 0.656; Ν = 5/ group  Main effect of lactation: F (1) = 1.559; Ρ = 0.230; Ν = 5/ group  Interaction treatment*lactation: F (1) = 0.013; Ρ = 0.911; Ν = 5/ group |
| Number of intersections | Mixed ANOVA | Main effect of treatment: F (1) = 0.023; Ρ = 0.884; Ν = 5/ group  Interaction treatment*radius: F (2.312) = 0.634; Ρ = 0.563; Ν = 5/ group |

## **Supplementary Table 8. Summary of main statistical parameters for all conducted experiments.**

|  | | **Sample size/condition** | | |  | |
| --- | --- | --- | --- | --- | --- | --- |
| **Mouse model** | **Experiment** | **Control** | | **Poly I:C** | **Parameter/Area** | **Statistics** |
| C57Bl/6N WT | Litters’ information | 17 mothers | | 16 mothers | Litter size | Student’s t test  t (31) = -0.028; Ρ = 0.978 |
|  |  | 17 mothers | | 17 mothers | Pups weight at PD4 | Student’s t test  t (32) = -0.437; Ρ = 0.665 |
|  |  | 6 mothers | | 6 mothers | Pups sex ratio | Mann Whitney U test  U=9; Ρ = 0.180 |
| C57Bl/6N WT | Mothers’ information | 16 mothers | | 16 mothers | Mothers’ weight gain | Mixed ANOVA  Interaction treatment*weight: F (1.544) = 0.095; *P* = 0.861  Main effect of treatment: F (1) = 0.257; Ρ = 0.616 |
|  |  | 11 mothers | | 10 mothers | Access to food | Student’s t test  t (21) = 0.387; P = 0.703 |
|  |  | 12 mothers | | 13 mothers | Access to water | Student’s t test  t(16.123) = -1.154; P = 0.265 |
| C57Bl/6N WT | Pup retrieval | 16 mothers | | 16 mothers | % of pups retrieved after 40’’ | Mann Whitney U test  U=64.5; Ρ = 0.015 |
|  |  |  |  |  | Latency | Student’s t test  t (20.290) = -2.891; Ρ = 0.009 |
|  |  | 16 mothers | | 15 mothers | nr of sniffs | Student’s t test  t (29) = 0.081; Ρ = 0.936 |
|  |  | 16 mothers | | 15 mothers | Relative failure index | Student’s t test  t (14.995) = -2.125; Ρ = 0.037 |
|  |  | 32 mothers | | | Retrieval time vs relative failure index | Spearman-Rho correlation  r_s_=0.897; Ρ < 0.001 |
|  |  | 15 mothers | | 15 mothers | nr of rearings | Student’s t test  t (19.858) = -2.392; Ρ = 0.027 |
|  |  | 30 mothers | | | Retrieval time vs nr of rearings | Spearman-Rho correlation  r_s_=0.593; Ρ = 0.001 |
| C57Bl/6N WT | USVs recording | 4 pups/ litter, 10-11 mothers | 4 pups/ litter, 9-10 mothers | | Amplitude | Student’s t test  t (18) = -0.936; Ρ = 0.362 |
|  |  |  |  |  | Duration | Student’s t test  t (19) = 1.436; Ρ = 0.156 |
|  |  |  |  |  | Frequency | Student’s t test  t (18) = 0.664; Ρ = 0.515 |
|  |  |  |  |  | Nr calls | Student’s t test  t (18) = -0.758; Ρ = 0.458 |
|  |  |  |  |  | Analysis of different call types | See Supplementary Table 5 |
| C57Bl/6N WT | mPOA neuronal reconstruction multipolar neurons | 4-5 neurons/ brain, 2 sections, 6 mothers and  6 nulliparous | | 4-5 neurons/ brain, 2 sections, 6 mothers and  6 nulliparous | Cumulative length | Two-way ANOVA  Treatment*lactation: F (1) = 7.888; Ρ = 0.011  Pairwise comparison  in mothers: poly I:C vs Vehicle, Ρ = 0.013  in nulliparous: poly I:C vs vehicle, Ρ = 0.228  in vehicle: mothers vs nulliparous, Ρ = 0.001  in poly I:C: mothers vs nulliparous, Ρ = 0.947 |
|  |  |  |  |  | Number of nodes | Two-way ANOVA  Treatment*lactation: F (1) = 4.654; Ρ = 0.043  Pairwise comparison  in mothers: poly I:C vs vehicle, Ρ = 0.056  in nulliparous: poly I:C vs vehicle, Ρ = 0.317  in vehicle: mothers vs nulliparous, Ρ = 0.008  in poly I:C: mothers vs nulliparous Ρ = 0.897 |
|  |  |  |  |  | Cell body area | Two-way ANOVA  Main effect of treatment: F (1) = 3.952; Ρ = 0.061  Main effect of lactation: F (1) = 10.180; Ρ = 0.005  Interaction treatment*lactation: F (1) = 2.763; Ρ = 0.112 |
|  |  |  |  |  | Sholl analysis | Mixed ANOVA  Interaction treatment*radius: F (2.490) = 2.686; Ρ = 0.077  Main effect of treatment: F (1) = 10.511, Ρ = 0.009 |
|  | mPOA pilot analysis pyramidal neurons | 6 neurons/ group | | 8-9 neurons/ group |  | See Supplementary Table 6 |
|  | mPOA pilot analysis bipolar neurons | 8-9 neurons/ group | | 6 neurons/ group |  | See Supplementary Table 6 |
| Gal::Cre//FlexEYFP | c-Fos staining | 2 60X images/ section, 2 sections, 5 mothers | | 2 60X images/ section, 2 sections, 6 mothers | mPOA | Student’s t test  Percentage Gal^+^ 🡪 t (9) = -0.518, Ρ = 0.617  Percentage c-Fos^+^/Gal^+^ 🡪 t (9) = 2.769, Ρ = 0.022 |
|  |  |  |  |  | vBNST | Student’s t test  Percentage Gal^+^ 🡪 t (10) = 0.289, Ρ = 0.778  Percentage c-Fos^+^/Gal^+^🡪 t (10) = -0.727, Ρ = 0.484 |
| Gal::Cre | Morphological analysis | 1 2x3 stitched image, 2 sections, 3 nulliparous | |  |  |  |
| Gal::Cre//vGat::Flp  Gal::Cre//vGlut2::Flp | AAV tracing | 1 2x3 stitched image, 2 sections, 2 nulliparous/ mouse line | |  |  |  |
|  | AAVrg tracing | 1 2x3 stitched image, 2 sections, 1 nulliparous/ mouse line | |  |  |  |
| C57Bl/6N WT | VTA spines density analysis | 16 dendritic segments/ brain, 2 sections, 5 mothers and 5 nulliparous | | 16 dendritic segments/ brain, 2 sections, 5 mothers and 5 nulliparous |  | See Supplementary Table 7 |
| C57Bl/6N WT | VTA neuronal reconstruction | 6 neurons/ brain, 2 sections, 5 mothers and  5 nulliparous | | 6 neurons/ brain, 2 sections, 5 mothers and  5 nulliparous |  | See Supplementary Table 7 |
| C57Bl/6N WT | Single unit in vivo electrophysiology | 21 neurons, 5 mothers | | 20 neurons, 5 mothers | Baseline  narrow spiking neurons | Mann Whitney U test  U = 122; Ρ = 0.022 |
|  |  | 16 neurons, 5 mothers | | 18 neurons, 5 mothers | Baseline  wide spiking neurons | Mann Whitney U test  U = 86; Ρ = 0.046 |
|  |  | 16 neurons, 5 mothers | | 18 neurons, 5 mothers | Baseline bursting % | Student’s t test  t (32) = -1.092; Ρ = 0.283 |
|  |  |  |  |  | Baseline spikes/bursts | Mann Whitney U test  U = 75.5; Ρ = 0.03 |
|  |  | 14 neurons, 4 mothers | | 6 neurons, 3 mothers | Pup retrieval  narrow spiking neurons | Mixed ANOVA  Interaction treatment*behavior: F (2) = 0.362; Ρ = 0.699  Main effect of treatment: F (1) = 4.230; Ρ = 0.055 |
|  |  | 15 neurons, 4 mothers | | 16 neurons, 3 mothers | Pup retrieval  wide spiking neurons | Mixed ANOVA  Interaction treatment*behavior:  F (2) = 4.954; Ρ = 0.01  Pairwise comparison  in post interaction: poly I:C vs vehicle, Ρ = 0.002  in poly I:C: approach vs post interaction, Ρ < 0.001  in poly I:C: pup retrieval vs post interaction, Ρ = 0.002 |

# **References**

1. Kentner A, Bilbo S, Brown A, Hsiao EY, Mcallister A, Meyer U, et al. Maternal immune activation: reporting guidelines to improve the rigor, reproducibility, and transparency of the model. 2019 Jan;245–58.

2. Arsenault D, St-Amour I, Cisbani G, Rousseau L-S, Cicchetti F. The different effects of LPS and poly I:C prenatal immune challenges on the behavior, development and inflammatory responses in pregnant mice and their offspring. Brain Behav Immun. 2014;38:77–90.

3. Gandhi R, Hayley S, Gibb J, Merali Z, Anisman H. Influence of poly I:C on sickness behaviors, plasma cytokines, corticosterone and central monoamine activity: Moderation by social stressors. Brain Behav Immun. 2007;21(4):477–89.

4. Risher WC, Ustunkaya T, Alvarado JS, Eroglu C. Rapid Golgi analysis method for efficient and unbiased classification of dendritic spines. PLoS One. 2014;9(9):e107591.
