# Supplementary material for: Gestational immune activation disrupts hypothalamic neurocircuits of maternal care behavior
Source: Mol Psychiatry. 2022 May 17;29(4):859–73. doi: 10.1038/s41380-022-01602-x (PMC9112243; doi:10.1038/s41380-022-01602-x)
Supplement: Supplementary file 3 — Supplementary Figure Legends [file 41380_2022_1602_MOESM3_ESM.docx]

# **Supplementary Figure** **Legends**

## **Supplementary Figure 1. MIA is not affecting pup USVs**

**A** Exemplary depiction of recorded pup USVs (scale bar: 50ms). **B-E** USVs in pups born from Poly I:C treated and control mothers (N = 9-11 mothers/group): **B** mean amplitude, **C** mean duration, **D** mean frequency, **E** number of calls per litter **F-K** Different types of USVs (classified according to Supplementary Table 1) in pups born from Poly I:C treated and control mothers (N = 9-11 mothers/group). 4 pups per litter were tested and considered for analysis. All data are presented as mean ± SEM, * Ρ < 0.05.

## **Supplementary Figure 2. Poly I:C injection is not affecting the morphology of mPOA pyramidal and bipolar neurons**

**A-C** Pilot analysis showing no difference in reconstructed bipolar neurons in cumulative length (N= 6-8 neurons/group), number of nodes (N = 6-9 neurons/group) and cell body area (N = 6,8 neurons/group) between MIA and controls. **D-F** Pilot analysis showing no difference in reconstructed pyramidal neurons in cumulative length (N = 6-8 neurons/group), number of nodes (N = 6-8 neurons/group) and cell body area (N = 6-9 neurons/group) between MIA and controls. All data are presented as mean ± SEM.

## **Supplementary Figure 3. Poly I:C-induced signalling cascade**

Graphical depiction of molecular elements of the intracellular pathway involved in Poly I:C signalling (1). Proteins identified in Gal^+^ clusters of mPOA neurons according to scRNA seq analysis (2) are highlighted in red. Proteins represented correspond to selected genes: *Il6r*, *Il1r1*, *Jak1/2*, *Stat1-6*, *Ifnar1/2*, *Tlr3*, *Ticam1*, *Tirap*, *Mal*, *Ikbkb*, *Chuk*, *nfk-b.* (Made with Biorender.com).

**Supplementary Figure 4.** **Representative brain section of a *Gal::Cre* female showing the area of injection in the VTA.**

The white arrow indicates the tract of the needle where AAVrg-FLEx-CAG-tdTomato was injected (20x magnification; scale bar: 1 000 µm).

## **Supplementary Figure 5. Morphology of VTA neurons is not altered in postpartum females after gestational Poly I:C injection**

**A** Magnification of Golgi-Cox impregnated dendritic segment with spines (100X magnification; scale bar: 10µm). **B-G** Poly I:C injected mothers do not show any alteration in dendritic spines density compared to vehicle injected mothers and nulliparous controls (N = 5 animals/group): **B** total dendritic spines density **C** density of *filopodia* **D** density of *mushroom* spines **E** density of *stubby* spines **F** density of *thin* spines **G** density of *long thin* spines. The number of spines was counted in four dendritic segments from 2/3 neurons per brain hemisphere for a total of 16 dendritic segments per brain. **H-I** Poly I:C injection does not result in alterations of **H** dendritic cumulative length or **I** number of dendritic nodes in either mothers or nulliparous controls (N = 5 animals/group). The morphology of 6 neurons was reconstructed from two sections per animal for a total of 30 neurons per group. All data are presented as mean ± SEM.

## **Supplementary Figure 6. Single-cell RNA sequencing identifies Gal^+^ clusters in the MPN that co-express cytokines and chemokine receptors.**

Sex-specific expressional profile of cytokine and chemokine receptors in the Gal^+^ clusters i8, i16 and i18 of the MPN; 0: females; 1: males (2).

**References**

1. Matsumoto M, Seya T. TLR3: interferon induction by double-stranded RNA including poly(I:C). Adv Drug Deliv Rev. 2008;60(7):805–12.

2. Moffitt JR, Bambah-Mukku D, Eichhorn SW, Vaughn E, Shekhar K, Perez JD, et al. Molecular, spatial, and functional single-cell profiling of the hypothalamic preoptic region. Science. 2018;362(6416):eaau5324.
