# Supplementary figures and images for: Gestational immune activation disrupts hypothalamic neurocircuits of maternal care behavior
Source: Mol Psychiatry. 2022 May 17;29(4):859–73. doi: 10.1038/s41380-022-01602-x (PMC9112243; doi:10.1038/s41380-022-01602-x)

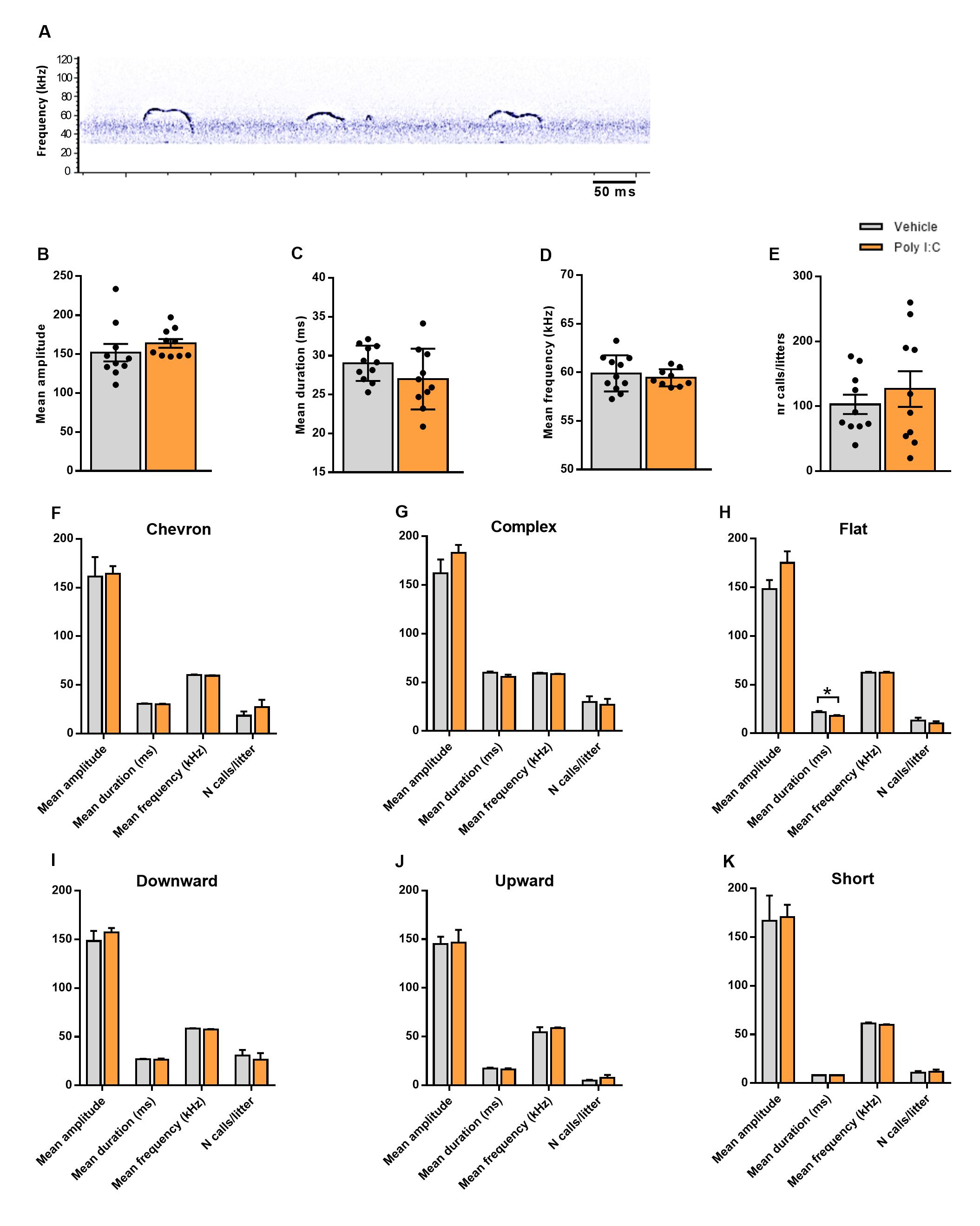

Supplement: Supplementary file 4 — Supplementary Figure 1 [file 41380_2022_1602_MOESM4_ESM.jpg]

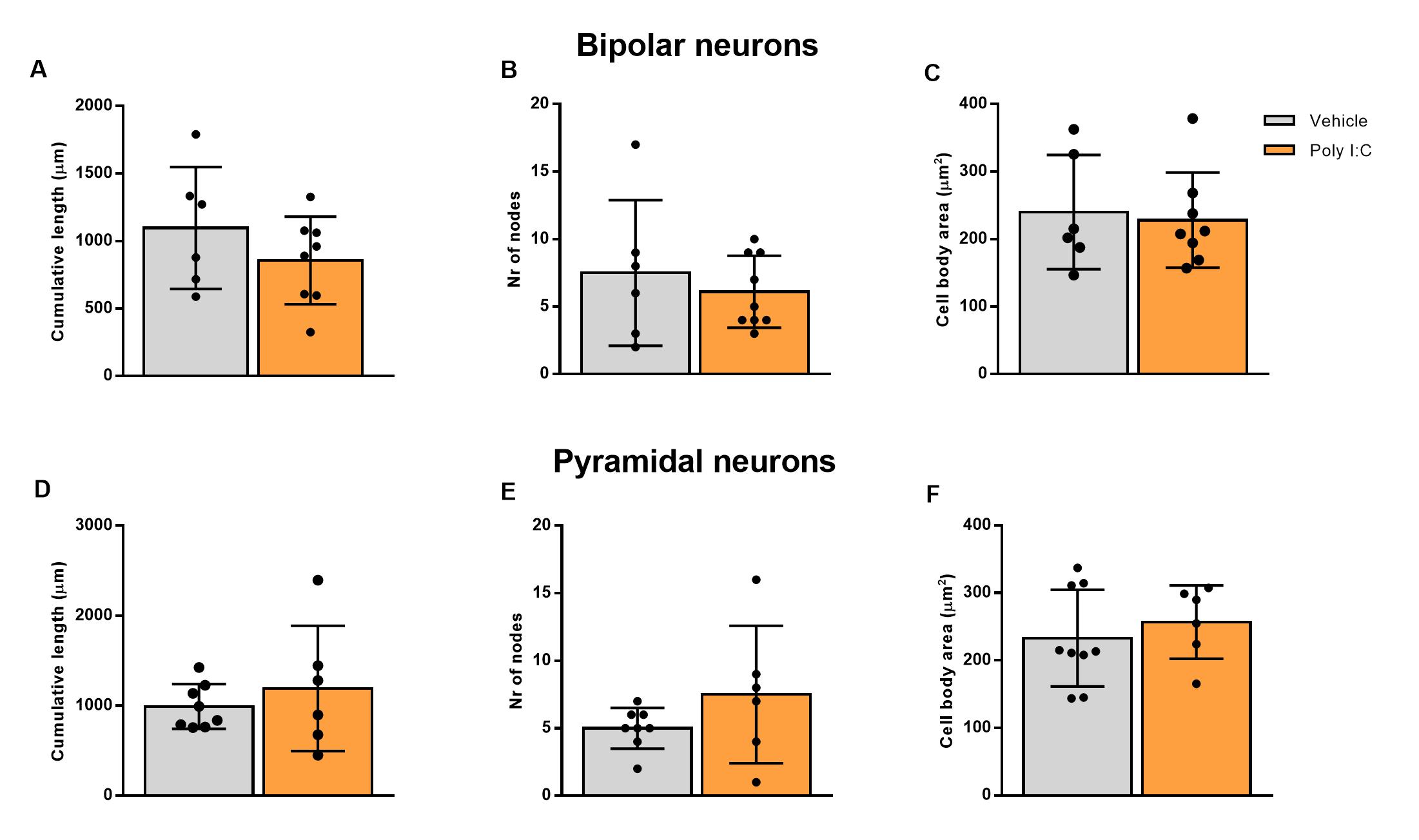

Supplement: Supplementary file 5 — Supplementary Figure 2 [file 41380_2022_1602_MOESM5_ESM.jpg]

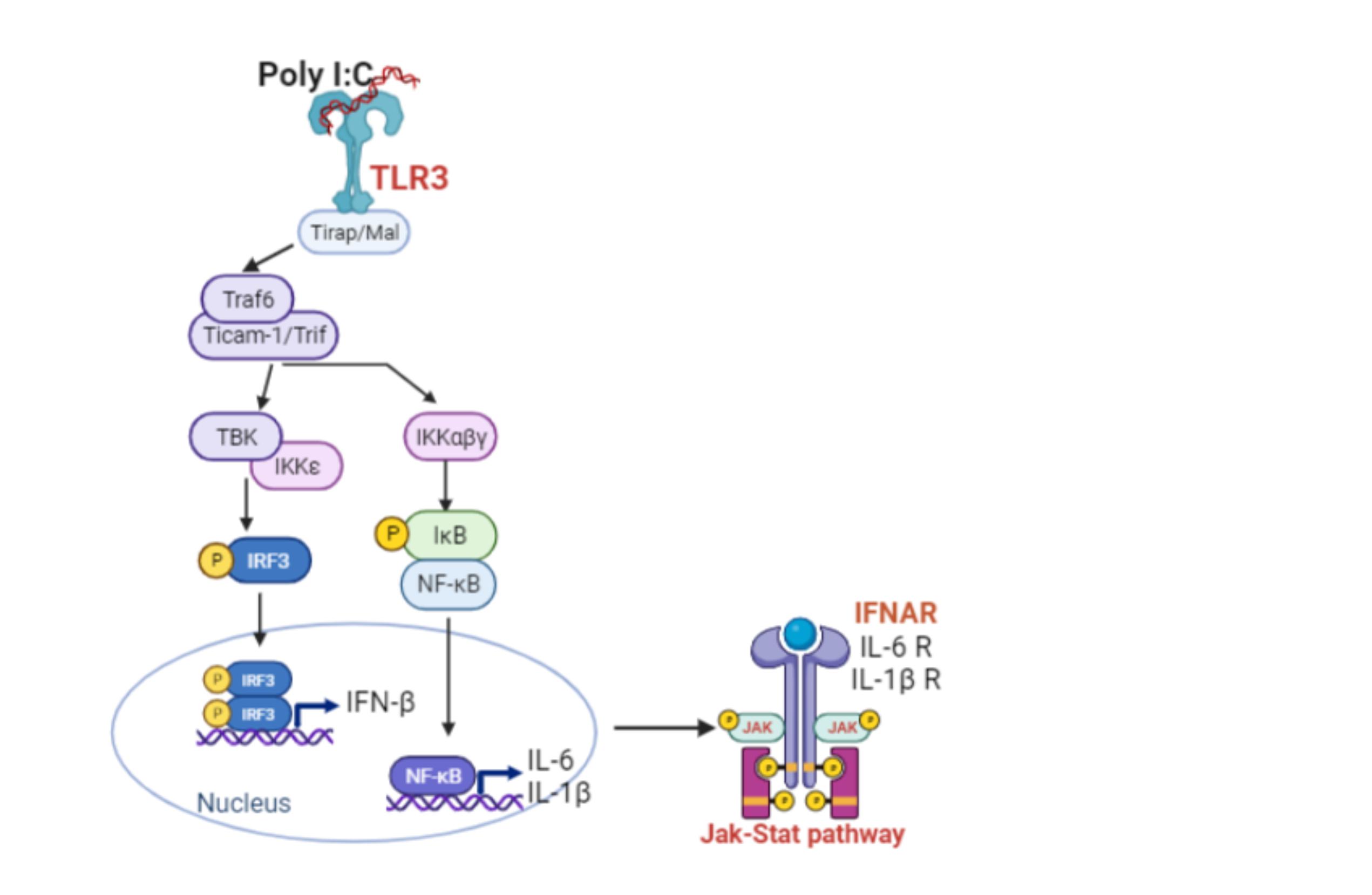

Supplement: Supplementary file 6 — Supplementary Figure 3 [file 41380_2022_1602_MOESM6_ESM.jpg]

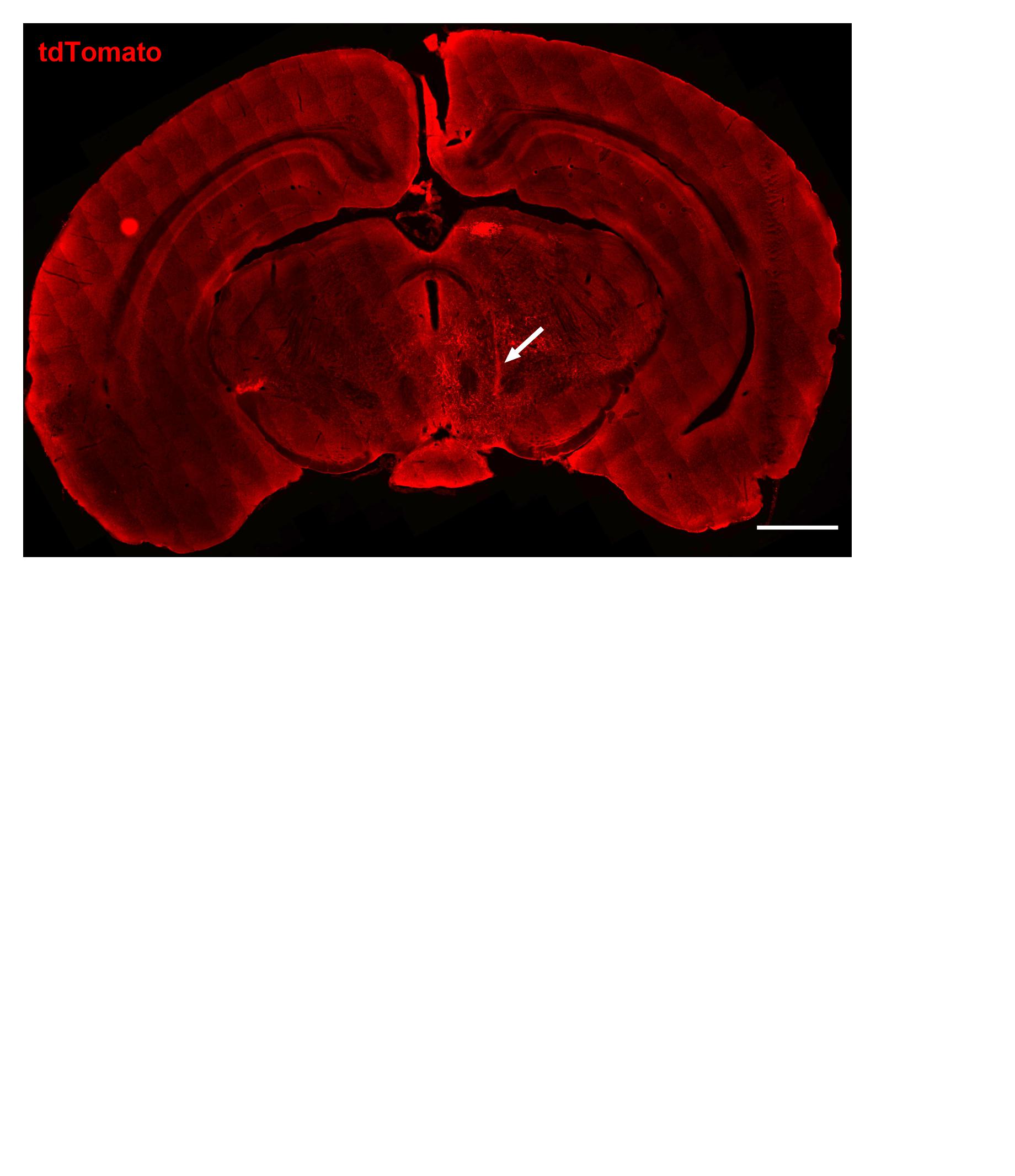

Supplement: Supplementary file 7 — Supplementary Figure 4 [file 41380_2022_1602_MOESM7_ESM.jpg]

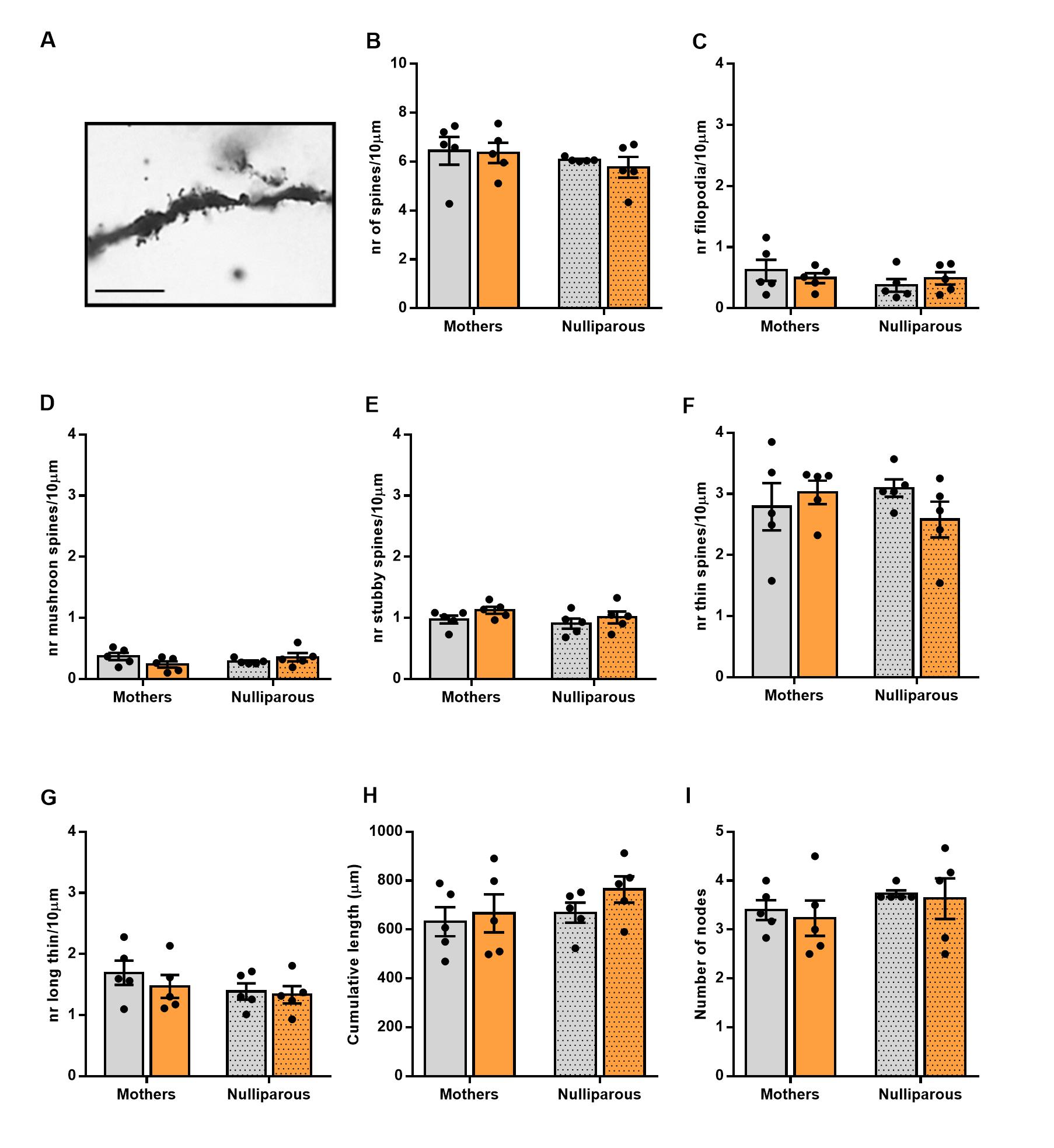

Supplement: Supplementary file 8 — Supplementary Figure 5 [file 41380_2022_1602_MOESM8_ESM.jpg]

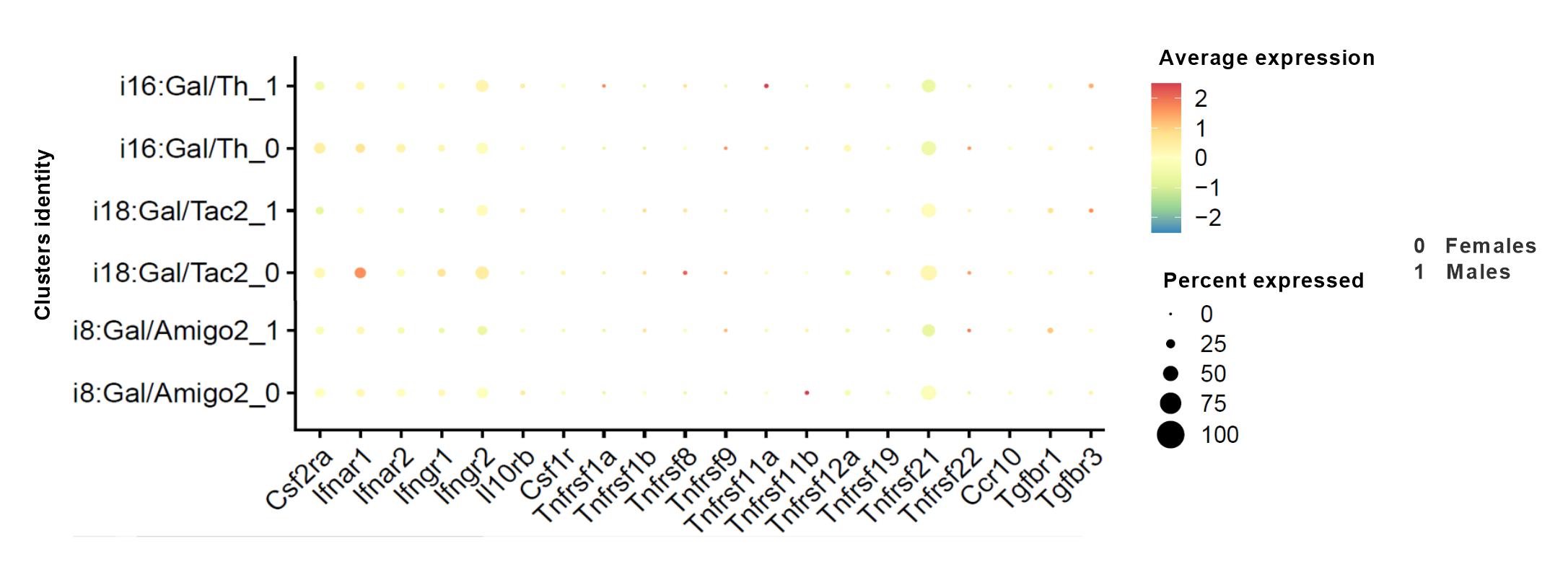

Supplement: Supplementary file 9 — Supplementary Figure 6 [file 41380_2022_1602_MOESM9_ESM.jpg]
